# Characterization of Cystatin B Interactome in Saliva from Healthy Elderly and Alzheimer’s Disease Patients

**DOI:** 10.3390/life13030748

**Published:** 2023-03-10

**Authors:** Cristina Contini, Simone Serrao, Barbara Manconi, Alessandra Olianas, Federica Iavarone, Giulia Guadalupi, Irene Messana, Massimo Castagnola, Carlo Masullo, Alessandra Bizzarro, Christoph W. Turck, Giuseppina Maccarrone, Tiziana Cabras

**Affiliations:** 1 Department of Life and Environmental Sciences, University of Cagliari, 09124 Cagliari, Italy; 2 Department of Basic Biotechnological Sciences, Intensive and Perioperative Clinics, Università Cattolica del Sacro Cuore, 00168 Rome, Italy; 3 Policlinico Universitario “A. Gemelli” Foundation IRCCS, 00168 Rome, Italy; 4 Istituto di Scienze e Tecnologie Chimiche “Giulio Natta”, Consiglio Nazionale delle Ricerche, 00168 Rome, Italy; 5 Proteomics Laboratory, European Center for Brain Research, (IRCCS) Santa Lucia Foundation, 00168 Rome, Italy; 6 Department of Neuroscience, Neurology Section, Università Cattolica del Sacro Cuore, 00168 Rome, Italy; 7 Proteomics and Biomarkers, Max Planck Institute of Psychiatry, 80804 Munich, Germany; 8 Department of Translational Research in Psychiatry, Max Planck Institute of Psychiatry, 80804 Munich, Germany

**Keywords:** cystatin B, saliva, alzheimer’s disease, interactome, affinity purification, mass spectrometry, bottom-up proteomics

## Abstract

Cystatin B is a small, multifunctional protein involved in the regulation of inflammation, innate immune response, and neuronal protection and found highly abundant in the brains of patients with Alzheimer’s disease (AD). Recently, our study demonstrated a significant association between the level of salivary cystatin B and AD. Since the protein is able to establish protein-protein interaction (PPI) in different contexts and aggregation-prone proteins and the PPI networks are relevant for AD pathogenesis, and due to the relevance of finding new AD markers in peripheral biofluids, we thought it was interesting to study the possible involvement of cystatin B in PPIs in saliva and to evaluate differences and similarities between AD and age-matched elderly healthy controls (HC). For this purpose, we applied a co-immunoprecipitation procedure and a bottom-up proteomics analysis to purify, identify, and quantify cystatin B interactors. Results demonstrated for the first time the existence of a salivary cystatin B-linked multi-protein complex composed by 82 interactors and largely expressed in the body. Interactors are involved in neutrophil activation, antimicrobial activity, modulation of the cytoskeleton and extra-cellular matrix (ECM), and glucose metabolism. Preliminary quantitative data showed significantly lower levels of triosophosphate isomerase 1 and higher levels of mucin 7, BPI, and matrix Gla protein in AD with respect to HC, suggesting implications associated with AD of altered glucose metabolism, antibacterial activities, and calcification-associated processes. Data are available via ProteomeXchange with identifiers PXD039286 and PXD030679.

## 1. Introduction

Cystatin B is a single chain protein of 98 residues belonging to the cystatin family, the largest group of endogenous cathepsin inhibitors [1]. Together with cystatin A, it forms the type-1 cystatin subfamily, sharing the same 3D structure, 80% sequence homology, and 52% identity [2]. Cystatin B, widely expressed in human tissues and cells, is able to inhibit cathepsin B, H, and L [1]. Cystatin A and B play important roles in the regulation of inflammation [3] and in the innate immune response [4]. Numerous additional functions have been proposed for cystatin B, including cell homeostasis maintenance, reduction of oxidative stress [5,6], participation in the process of autophagy [7], prevention of apoptosis [8], and a neuronal protective role [3,5]. Cystatin B subcellular localization can also vary according to the state of cell differentiation. It has been shown to be mainly nuclear in proliferating cells and cytoplasmic and lysosomal in differentiated cells. In addition, the cellular localization of cystatin B varies with the cellular processes it is involved in [9].

Mutations in the cystatin B gene cause the development of the most common form of progressive myoclonus epilepsy [10]. Moreover, it was proposed that cystatin B could be involved in the imbalance of the extracellular environment in the brain, which is considered one of the causes contributing to the development of several neurological and psychiatric disorders [11]. Mainly by virtue of its cathepsin inhibitory activity, indeed, unrestrained proteolysis because of an imbalance between active proteases and their endogenous inhibitors has been associated with neuronal cell death in different neuronal diseases, such as brain tumors, stroke, epilepsy, Alzheimer’s disease (AD), and neurological autoimmune diseases [12]. Altered levels of cystatin B have been observed in the context of AD, where high amounts of the protein were found post-mortem in the brains of patients [13]. Our recent studies on the salivary proteome in AD patients evidenced significantly higher levels of cystatin B compared to age-matched elderly healthy individuals [14,15], suggesting the existence of a self-protection mechanism in the patients, which could reflect the neuronal protective role of cystatin B in the brain [14,15]. AD is the most common neurodegenerative disease (ND) afflicting the elderly population, and its principal hallmarks are misfolded beta-amyloid (Aβ) and tau proteins in the central nervous system (CNS) [16]. AD is a complex disease that affects individuals differently and has a multifactorial etiology. Many environmental and genetic factors can contribute to the onset and progression of the disease. The final and definite diagnosis of AD is still linked to a post-mortem neuropathological study showing the presence of abundant and diffuse beta-amyloid plaques and neurofibrillary tangles, as well as diffuse and large neuronal loss. Many clinical diagnostic tools supporting the clinical diagnosis are available, such as cerebrospinal fluid (CSF) studies, detection and measurement of beta-amyloid and tau protein levels, PET brain studies with specific radioligands for beta-amyloid and tau, and brain-magnetic resonance imaging (MRI) [17]. All these diagnostic tools are limited by their costs (PET- and brain-MRI), and complexity, or invasiveness (CSF study). Many peripheral diagnostic biomarkers have been proposed, but still none have shown to be sensitive and specific enough to be considered good candidates [18,19,20]. Therefore, it is a global challenge to find novel, peripheral potential biomarkers useful for developing effective screening methods for large-scale application.

Recent studies have shown a significant association between AD and protein-protein interactions (PPIs), involving several aggregation-prone proteins besides Aβ and tau [21,22,23]. It was suggested that the aggregation propensity of specific proteins associated with NDs is influenced by interactions with other proteins [22], and that protein interaction networks may play a central role in both driving and mitigating inclusion formation [23]. Therefore, identifying, and characterizing PPIs can provide valuable knowledge on the formation of aberrant protein aggregates and the cellular processes that control them, as well as on the mechanisms of disease. Major contributions to the investigation of PPI networks associated with NDs have been made by proteomics studies that rely on protein co-immunoprecipitation (CoIP) experiments, including affinity purification mass spectrometry (AP-MS) or yeast-two-hybrid assays, methods that are able to detect not only high-affinity PPIs but also weak, transient interactions [22,23]. However, the function of the aggregation-prone proteins and the complexity of the PPIs and their networks in ND pathogenesis, especially in AD, remain to be fully elucidated.

Several studies have demonstrated the ability of cystatin B to interact with other proteins [24,25,26] and to form a multiprotein complex in the rat cerebellum [27]. In vitro studies have shown that cystatin B oligomers can inhibit or facilitate Aβ fibril growth according to their size [25]. These properties of cystatin B, our previous insights on salivary proteoforms of cystatin B associated with AD [14,15], and the relevance of the characterization of peripheral potential biomarkers and of their interactome in AD led us to investigate the possible involvement of cystatin B in a PPI network in the oral cavity. The feasibility, non-invasiveness, painlessness, and low-cost of collecting saliva make this biofluid an optimal choice to discover protein biomarkers useful for the diagnosis and risk assessment of several diseases, including AD [28,29], as opposed to blood and CSF [20]. Saliva contains proteins/peptides of both glandular and non-glandular origin, as well as proteins from blood and the CNS, and changes in the salivary proteome may reflect pathological conditions at the CNS level. In addition, in AD patients, the function of the major salivary glands and their secretion can be altered [28,30,31]. Saliva is a biofluid that has been investigated in the AD field [14,15,28,30,31], and to date, several salivary biomarkers of AD have been studied by different approaches, even if with contrasting outcomes [20,28,31,32].

Based on these premises, this study focused on the search and characterization of PPIs of cystatin B in saliva from AD patients and evaluated differences and similarities in the composition and abundance of the interactome between AD and healthy control individuals. For this purpose, we used an AP-MS method, performing CoIP experiments coupled to bottom-up proteomics analysis. Furthermore, possible significant differences in the composition of the interactome between AD and healthy control subjects could be under consideration as a potential salivary peripheral AD biomarker.

## 2. Materials and Methods

### 2.1. Study Subjects

**Figure 1:**
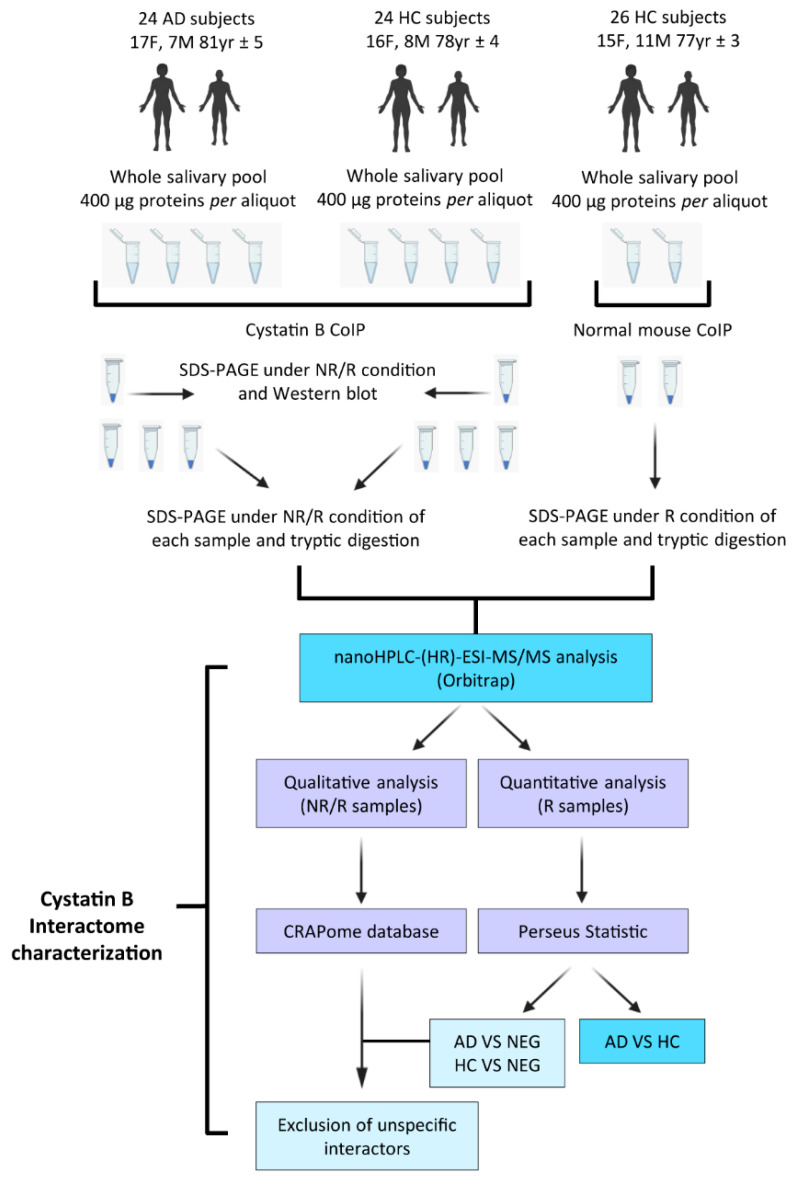
Experimental design and workflow.

Twenty-four subjects affected by AD were recruited at the Neurology Department of the “Fondazione Policlinico Universitario A. Gemelli”, Catholic University of the Sacred Heart, Rome (17 females and 7 males; 81 yr ± 5; mean age ± standard deviation (SD)). Thirty-four volunteers were enrolled as healthy controls; twenty-four healthy control subjects were considered the positive control group (HC) to be compared with the AD group; it was composed of 16 females and 8 males (78 yr ± 4; mean age ± SD) chosen to match the AD group in number, sex, and age; twenty-six HC subjects were considered the experimental negative control (NEG) in the AP-MS experiments. The NEG group was composed of 15 females and 11 males (77 yr ± 3; mean age ± SD). To increase the heterogeneity of the NEG group, sixteen subjects (11 females and 5 males) were the same as those included in the HC control group, and ten subjects (4 females and 6 males) were not included in the HC group. The schematic summary of the experimental design applied in this study to characterize the salivary interactome of cystatin B is shown in Figure 1. Demographic features of the subjects are shown in Table 1. Donors, either healthy volunteers or patients, were non-smokers and did not show pathological alterations of the oral cavity during saliva collection. The informed consent process was based on the latest stipulations established by the Declaration of Helsinki, and the study was approved by the formal ethical committee of the Catholic University of the Sacred Heart, Rome (protocol number 3457). The AD diagnosis was determined according to standardized criteria [33] and included both moderate (n = 8) and mild (n = 16) AD patients. Patients’ pharmacological treatment and clinical features are reported in supplementary Table S1.

### 2.2. Sample Collection and Treatment

Samples of non-stimulated whole saliva were collected between 9:00 and 12:00 a.m. Donors, in fasting conditions, were invited to sit in a relaxed position and to swallow. Whole saliva was collected as it flowed into the anterior floor of the mouth with a soft plastic aspirator for less than 1 min and transferred to a plastic tube cooled on ice. All samples were immediately diluted in a 1:1 *v*/*v* ratio with PBS (270 mM NaCl, 5 mM KCl, 20 mM NaHPO_4_, and 4 mM KH_2_PO_4_) containing cOmplete™, EDTA-free protease inhibitor cocktail (Sigma-Aldrich/Merck, Darmstadt, Germany), and gently centrifuged at 1000 g for 5 min at 4 °C before being stored at −80 °C until further analysis. The total protein concentration (TPC) was determined in duplicate for each sample by the bicinchoninic acid (BCA) assay kit (Sigma-Aldrich/Merck), following the manufacturer’s instructions. For several samples, the collected volume of whole saliva (under 100 μL) as well as the TPC were very low (Table 1), thus not usable for single CoIP experiments. To obtain CoIPs comparable between the AD and HC groups, we decided to pool the salivary samples. Three salivary pools were prepared: from 24 AD samples (AD pool, 0.95 μg/μL, total protein amount 1.6 mg), 24 HC samples (HC pool, 0.46 μg/μL, total protein amount 1.6 mg), and from 26 HC samples (NEG pool, 1.23 μg/μL, total protein amount 0.8 mg) (Table 1). To prepare the three pools, different volumes were used from each sample on the basis of their TPC (supplementary Table S2), so that each sample contributed the same amount of protein, 66.67 μg in the case of both the AD and HC pools and 30.77 μg for the NEG pool. Four aliquots with 400 μg of total protein amount from both the AD and HC pools and two aliquots with 400 μg of total protein amount from the NEG pool were prepared for CoIP experiments.

### 2.3. Affinity Purification

CoIP experiments were performed using 100 μL of SureBeads™ Protein G Magnetic Beads (Bio-Rad, Hercules, CA, USA) optimized for 400 μg of total proteins per experiment. Beads were first magnetized to discard their store-solution and then washed 3 times by resuspension in 1 mL PBS buffer containing 0.1% Tween-20 (Sigma-Aldrich/Merck, PBS-T). For AD and HC pools, beads were resuspended in 200 μL PBS-T containing 4 μg cystatin B mouse monoclonal antibody (Invitrogen/Thermo-Fisher Scientific, Waltham, MA, USA) or, for the NEG pool, 4 μg of normal mouse IgG polyclonal antibody (Sigma-Aldrich/Merck) and gently rotated for 10 min at room temperature (RT). To remove unbound antibody, the beads were magnetized and washed again 3 times with PBS-T. Salivary pools were added to the beads-antibody complex and gently rotated for 1 h at RT. Non-specifically bound proteins were removed by washing 3 times with PBS-T. Finally, the immunoprecipitated (IP) samples were collected by resuspension of the beads in 50 μL 60 mM Tris/HCl pH 6.8 containing 2% SDS and incubation for 10 min at 70 °C. Before any further analysis, all IP samples were quantified by BCA assay in duplicate using a NanoDrop 2000 Spectrophotometer (Sigma-Aldrich/Merck). IPs obtained from one aliquot of both AD and HC pools were analyzed by western blot; IPs obtained from three aliquots of both AD and HC pools and from two aliquots of the NEG pool were analyzed by SDS-PAGE and tryptic digestion.

### 2.4. Western Blot

AD and HC IPs were mixed 1:1 (*v*/*v*) with 60 mM Tris/HCl pH 6.8 containing 2% SDS, 20% glycerol, and 0.02% bromophenol blue with or without 2% 2-mercaptoethanol to perform SDS-PAGE under reducing (R) and non reducing (NR) conditions, respectively, using 4.5 μg of total protein for each IP. Electrophoretic separation was performed with the Mini-PROTEAN Tetra cell (Bio-Rad) at 180 V and Precision Plus Protein™ WesternC™ Blotting Standards (Bio-Rad) were used as molecular weight standards. Proteins were transferred to 0.2 μm PVDF membranes according to the manufacturer’s instructions for the Trans-blot Turbo system (Bio-Rad). After the transfer, PVDF membranes were equilibrated for 1 h with blocking solution (5% blotting-grade blocker, Bio-Rad, in TBS containing 0.05% Tween-20, TBS-T), and then for 1 h under stirring with cystatin B mouse monoclonal primary antibody (Invitrogen/Thermo-Fisher Scientific) diluted 1:1000 with TBS-T. After 5 × 5 min washing with TBS-T, membranes were incubated for 1 h with the anti-mouse secondary Ab (HRP conjugated dil. 1:5000 in TBS-T). After 5 × 5 min washing with TBS-T, membranes were incubated with the detection solution (Clarity Max™ Western ECL Substrate, Bio-Rad). The detection of cystatin B positive signals was performed with the ChemiDoc MP Imaging System (Bio-Rad) and analyzed with Image Lab 4.0.1 software.

### 2.5. Tryptic Digestion

The NR and R IPs (12 μg/each, 6 μg per sample) and NEG IPs (6 μg/each) were separated by SDS-PAGE (Supplementary Figure S1). Electrophoretic separation was performed as previously described. The SDS-PAGE gels were stained with Bio-Safe TM Coomassie G250 stain (Bio-Rad), and then each lane was cut into slices. All gel slices were digested with trypsin and analyzed by high-resolution (HR)-MS. In total, MS analyses were performed on 20 samples from NEG IPs (2 × 10 gel slices for R samples), 78 samples from AD IPs (3 × 13 gel slices for a total of 39 for NR samples and 3 × 13 gel slices for a total of 39 for R samples), and 78 samples from HC IPs (3 × 13 gel slices for a total of 39 for NR samples and 3 × 13 gel slices for a total of 39 for R samples). Supplementary Figure S1 shows the SDS-PAGE gels obtained from AD, HC (panel A), and NEG (panel B) Ips and the number of gel slices. For de-staining and peptide extraction procedures, the protocol of Gundry et al. was applied [34]. Extraction was repeated twice and followed by drying under vacuum using a SpeedVac system (Sigma-Aldrich/Merck). The dried peptides were resuspended in 2% formic acid (FA; Thermo-Fisher Scientific, San Jose, CA, USA) and filtered with Corning^®^ Costar^®^ Spin-X^®^ plastic centrifuge tube filters and cellulose acetate membranes with a pore size of 0.22 μm (Sigma-Aldrich/Merck). The desalting of samples prior to HR-MS/MS analysis was performed with a Pierce C18 zip tip and 10 μL (Thermo-Fischer Scientific). The desalted peptides were lyophilized and stored at −20°C until the HR-MS/MS analysis.

### 2.6. Nano-HPLC-HR-MS/MS Analysis

The analyses of each sample were performed with a Q-Exactive Plus mass spectrometer (Thermo-Fisher Scientific), coupled to a Nano Spray Flex source, and connected to an Ultimate 3000R (Dionex, Sunnyvale, CA, USA) HPLC system. Reverse phase high performance liquid chromatography (RP-HPLC) was performed using a nanocolumn (150 mm × 75 µm inner diameter) pulled in-house (Puller P-1000, Sutter Instrument, Novato, CA, USA), packed with ReproSil-Pur C18 beads 1.9 μm (Dr. Maisch GmbH, Tübingen, Germany). Tryptic peptides were resuspended in solvent A, a 0.1% formic acid (FA) solution, and 5 μL were injected. Chromatographic separation of the peptides was achieved with solvent B (0.1% FA/95% acetonitrile (ACN) *v*/*v*, Thermo-Fisher Scientific) at a flow rate of 0.3 μL/min with the following gradient: 0–15 min to 2%; 15–125 min to 30%; 125–145 min to 60%; 145–146 min to 98%. For NEG IPs tryptic peptides, we used the following gradient: 0–15 min to 2% solvent B; 15–75 min to 30%; 75–80 min to 40%; 80–90 min to 98%. The MS operated in data dependent acquisition mode at 1.9 kV and 275 °C of capillary temperature. MS/MS analyses were performed at resolution 70.000, and the 10 most intense ions were considered for high-energy collisional dissociation (HCD) fragmentation. Spectra were acquired by Xcalibur software (Thermo-Fisher Scientific) and analyzed using Proteome Discoverer (PD, version 2.4, Thermo-Fisher Scientific) with the SEQUEST HT cluster search engine (University of Washington, licensed to Thermo Electron Corporation, San Jose, CA, USA) against the UniProtKB human database (188,453 entries, release 2019_03). MS data from corresponding SDS-PAGE lanes of the same triplicate samples were merged in the PD analyses with the following parameters: up to two missed tryptic cleavages; 10 ppm for peptide mass tolerance; and 0.6 Da for peptide and fragment ions; a FDR of 0.01 (strict) and 0.05 (relaxed). Set PTMs were: carbamidomethylation of cysteine as a stable modification; oxidation of methionine and tryptophan; serine/threonine phosphorylation; C-terminal pyroglutamic residue; N-terminal acetylation; and methionine loss as dynamic modifications. Peptides were filtered for high confidence and a minimum length of 6 amino acids; proteins were filtered for at least 2 unique identified peptides. For NR samples, the identification of a protein with only 1 unique peptide was accepted when the same protein was identified for R samples by at least 2 unique peptides. Contaminants from sample manipulation (keratins) were excluded, as were non-specifically bead binding proteins (i.e., hemoglobin and immunoglobulins) [35,36]. The label free quantification (LFQ) was performed only for R samples, and the variance of the protein abundances in each replicate of the AD and HC sample groups was compared by PD software. LFQ abundances were normalized against the total peptide amount in the precursor ion quantifier node of the PD software, and normalized data were used for statistics. All the results obtained by HR-MS/MS have been deposited to the ProteomeXchange Consortium (http://ww.ebi.ac.uk/pride (accessed on 5 November 2019)) via the PRIDE, version 2.5.2, [37] partner repository with the dataset identifiers PXD030679 and PXD039286.

### 2.7. Statistical Analysis

Statistics was performed with Perseus (version 1.6.15.0, Max-Planck-Institute of Biochemistry) [38], following the instructions provided for label-free interaction data. Protein LFQ intensities calculated by PD software were loaded as data matrices into Perseus and transformed into a Log2 scale. When a protein was not detected in one replicate or in one group, the LFQ value input in the matrix was “0.” Replicates were grouped by assigning a categorical factor so that the tool could recognize LFQ intensities of proteins from replicates belonging to the same group. Proteins were considered “valid” within Perseus only when measured in all replicates of the same group. The comparison between AD or HC proteins’ LFQ abundances with respect to NEG was carried out with the two-sample student t-test. The differences in the abundancies were considered significant when the *p* value was less than 0.05 and the fold change was above ±1.5 with 250 randomizations. The -Log10 of the *p* values and the Log2 of the fold change were utilized by Perseus for the analysis. The determination of non-specific interactors was assessed as follows: proteins were excluded when intensities were significantly higher in NEG IPs or unchanged between NEG and AD or NEG and HC. All identified proteins were further checked against the Contaminant Repository for Affinity Purification (CRAPome, version 2.0, free available at https://reprint-apms.org/ (accessed on 1 April 2016.)) [36] with Tandem Epitope Tag AP-MS as the experiment type. When a protein was observed in more than 25% of the reported experiments, it was excluded as a potential non-specific interactor. Therefore, the comparison between AD or HC proteins’ LFQ abundances was carried out with the two-sample student *t*-test, and the differences were considered significant when the *p* value was less than 0.05 and the fold change was above ±1.0 with 250 randomizations. Further, in this case, the -Log10 of the *p* values and the Log2 of the fold change were utilized by Perseus for the analysis.

### 2.8. Biological Processes and Tissue Enrichment Analyses

The list of identified cystatin B interactors was submitted to the ClueGO plugin (v. 2.5.8) via Cytoscape software (v. 3.9.1) [39]. A term functional analysis of enriched biological processes was performed using the enrichment right-sided hypergeometric test and Bonferroni step down statistical options. Only biological processes with *p* < 0.01 were accepted, with the kappa score set at 0.4. Minimum and maximum tree interval values were 3–7, the evidence code decision tree was set to “all,” and a minimum of 2 genes and 4% of genes were selected for GO terms. The Uniprot-KB code of cystatin B and its identified interactors were plotted on STRING version 11.5 (https://string-db.org/ (accessed on 12 August 2021)) to assess the network functional enrichment of tissue expression using the tool’s default parameters.

## 3. Results

The AP-MS approach applied in this study to detect and characterize possible salivary PPIs of cystatin B in AD and HC individuals was based on CoIP with cystatin B Ab. This procedure was able to co-immunoprecipitate the interactome of the salivary cystatin B, as demonstrated in the following subsections.

### 3.1. Immune-Detection of Cystatin B in IPs

**Figure 2:**
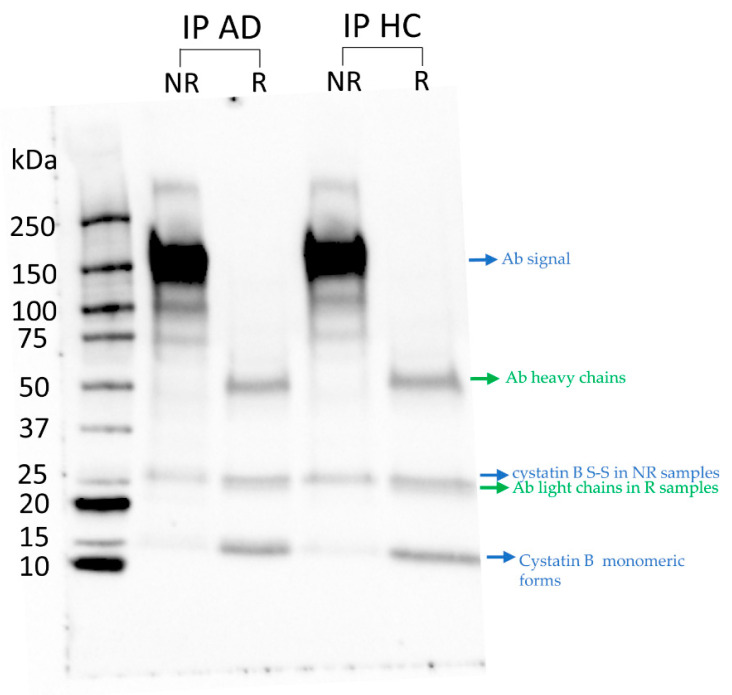
Western blot with cystatin B antibody after CoIP assays of whole saliva pools from 24 AD patients and 24 HC subjects (Table 1) in NR and R samples. Arrows indicate what each signal refers to.

The western blot analysis of the IPs obtained from the two salivary pools of AD and HC showed that the same positive signals were detectable for both groups (Figure 2). A positive signal was detected in NR samples with a molecular weight (MW) >250 kDa, which was not detected in R samples. The signal was attributed to a high molecular weight multiprotein complex associated in saliva with cystatin B, which showed to be resistant to SDS denaturing conditions but not to the reducing conditions. The presence of cystatin B in the slices at MW > 250 kDa (triple dots in Figure 2) was confirmed by bottom-up (HR)-MS/MS analysis (available on the ProteomeXchange repository with the PXD039286). Moreover, positive signals corresponding to different salivary proteoforms of cystatin B were detected and indicated in Figure 2 with a single dot (for the monomeric forms) and double dots (for the dimeric form). Indeed, under non reducing conditions, it was possible to detect signals at low MWs attributable to the Cys-modified proteoforms of cystatin B usually observed in adult human saliva [40], namely the cysteinylated and glutathionylated monomeric forms (Th. Mav, 11301 Da and 11487 Da, respectively) and the disulfide dimeric form (Th. Mav, 22.361 Da). The attribution of these Ab positive signals to cystatin B was confirmed by the bottom-up (HR)-MS/MS analysis (ProteomeXchange identifier PXD039286).

Under reducing conditions, only the signal attributed to the unmodified cystatin B was detected (Th. Mav: 11.181 Da) (ProteomeXchange identifier PXD039286), and it corresponded to the total cystatin B present in the IPs.

### 3.2. Identification of the Co-Immunoprecipitated Cystatin B Interactors

The bottom-up HR-MS/MS analysis of the proteins co-immunoprecipitated from the AD and HC salivary samples, and separated by SDS-PAGE resulted in the identification of 206 proteins for the R samples and 254 proteins for the NR samples, while 139 proteins were identified in the NEG IP samples. The (HR)-MS/MS results of their identification are available on the ProteomeXchange repository with the access code PXD030679. By excluding contaminant proteins, the number of identified proteins was reduced to 153 (R samples) and 190 (NR samples) in CoIPs from the AD and HC groups, and 86 (R samples) in IP samples from the NEG group (Supplementary Table S3). The same proteins were identified in AD and HC samples in R samples, as well as in NR samples (Supplementary Table S3). The most commonly characterized PTMs were the N-terminal acetylation (NTA) of the amino acid residue at position 1, and the loss of the N-terminal methionine (M_1_-loss), followed or not by NTA of the amino acid residue 2.

Two proteins identified in both R and NR samples showed different PTMs: heat shock protein beta-1 (HspB1; P04792) carrying phosphorylation at serine 82 in R samples and cellular retinoic acid-binding protein 2 (CRABP2; P29373) carrying a M_1_-loss, a modification not reported in the Uniprot-KB data bank (Supplementary Table S3), in NR samples. Another PTM not indicated in the data bank was the M_1_-loss followed by the NTA of the ADP/ATP translocase 2 (P05141).

The comparison of the protein LFQ abundance among AD, HC, and NEG groups for R samples (Supplementary Table S4) allowed the exclusion of unspecific interactors and kept only the proteins enriched in both AD and HC with respect to NEG samples. Supplementary Table S4 shows the enrichment of all interactors in the AD and HC groups with respect to NEG as a result of the student t-test and expressed by fold change and a −log10 *p* value for the two comparisons. These results are also shown in the Volcano plot in Supplementary Figure S2, where AD enriched proteins are represented by blue dots (A) and HC enriched proteins are represented by green dots (B). Grey dots in both panels represent unchanged proteins, while red dots are NEG enriched proteins. Further examination of the identified IP proteins for AD and HC in both R and NR samples was performed to exclude possible artificial interactors of the bait protein using CRAPome software (Supplementary Table S4).

On the basis of the exclusion criteria, only 82 proteins identified and quantified with accuracy in R samples (cystatin B included) have been further investigated, and they are shown in Table 2, which reports their UniProt-KB code and the molecular weight (MW) as obtained by PD software elaboration. The MW of the parent pro-protein is indicated when the sequence identified by the PD software cannot be attributed to a certain naturally occurring peptide, for example, in the case of the mature bioactive peptides derived from the precursor pro-proteins cathelicidin antimicrobial peptide (P49913) and neutrophil defensin 1 (P59665). In the case of cathelicidins, the sequenced tryptic fragments (Fr. 89–101; Fr. 108–118) identified the pro-domain known as the cathelin-like domain (CLD, 31–131 as position on the pro-protein precursor), so named because of the high degree of sequence homology to cathelin, a protein isolated from pig leukocytes and belonging to the cystatin family of cysteine protease inhibitors [41]. In the case of neutrophil defensin 1 precursor, the tryptic fragments (Fr. 70–78; Fr. 70–79; Fr. 80–88; Fr. 79–88) were common to the three bioactive peptides generated by the cleavage occurring during maturation of the pro-protein (α-defensin 1; α-defensin 2; HP 1–56), therefore it was not possible to distinguish them, which are indicated in the text generally as “peptides from neutrophil defensin 1 precursor.” It is to underline that, despite the sequence similarities between cystatin B and cystatin A, it was excluded that the monoclonal Ab anti-cystatin B we used could also recognize cystatin A as a bait protein. Indeed, the Ab was built against the entire sequence of cystatin B; moreover, if the antibody had recognized both cystatin B and A, we would expect to identify and measure comparable ratios of the two proteins under both R and NR conditions in all the samples. Instead, we identified cystatin A under NR conditions (Supplementary Table S3), but based on the criteria used to exclude unspecific interactors (NEG comparison and CRAPome score), cystatin A did not result in a candidate interactor of cystatin B among the 81 interactors presented under R conditions (Table 2).

**Figure 3:**
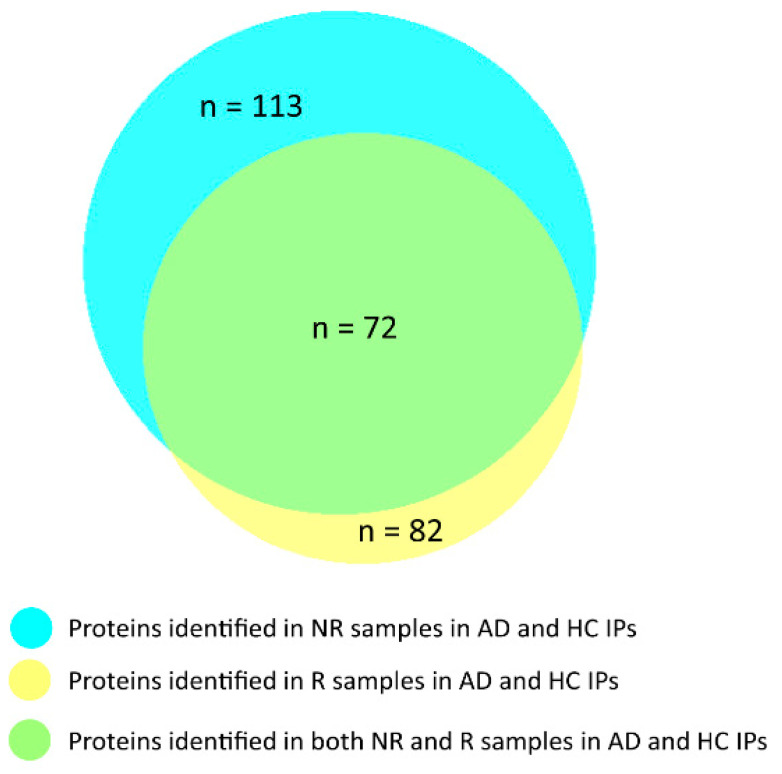
Venn diagram of the proteins identified in AD and HC IPs in NR and R samples.

A qualitative comparison between proteins identified in R and NR samples by the Venn diagram (Figure 3) shows that 72 out of 82 proteins (including cystatin B) identified in R samples were also characterized in NR samples. 10 proteins were identified only in R samples and 41 proteins only in NR samples. The information on the proteins distributed in the Venn diagram and their Uniprot-KB codes are reported in Supplementary Table S5.

Moreover, 52 proteins out of 81 cystatin B interactors identified in R samples, and cystatin B itself, were detected in the gel-slices above 150 kDa in NR samples, and they are indicated with a black dot in Table 2. This result confirmed the specificity of the positive cystatin B signal observed at molecular weight >250 kDa in the western blot (Figure 2) and suggests that the majority of identified protein interactors appear to be associated with the large protein complex and are resistant to denaturing but not reducing conditions during SDS-PAGE analysis.

### 3.3. Biological Pathway and Tissue Analyses

**Figure 4:**
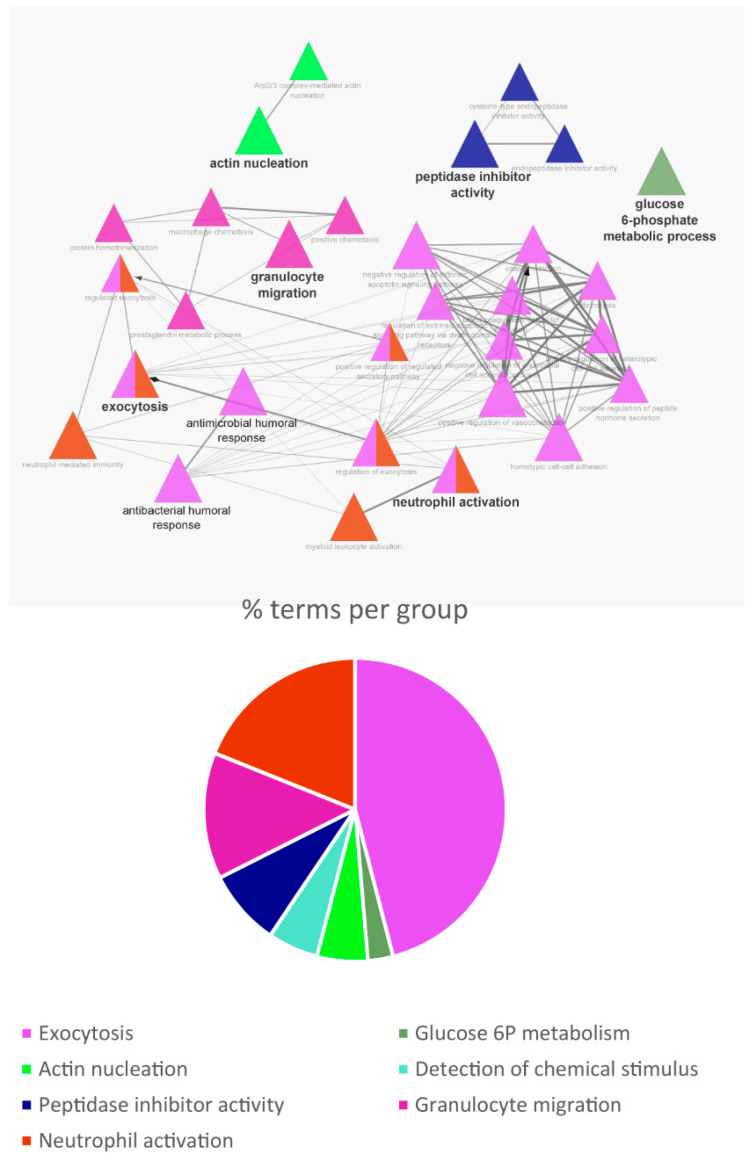
Biological processes enrichment analysis performed with the ClueGo plugin in Cystoscape.

For the pathway analysis, only the proteins identified in the R samples (#82, cystatin B included) were considered. The analysis of significant biological pathways in which cystatin B and its 81 interactors participate is shown in Figure 4. The data indicated that PPIs among these proteins involved the following biological processes: exocytosis, granulocyte migration, and neutrophil activation, with minor participation in actin nucleation, peptidase inhibitor activity, and glucose metabolism. The analysis performed by the STRING tool showed that the 82 interactors were ubiquitous proteins, although those typically localized in the blood system and in the oral cavity showed the best significance of the FDR. The detailed information obtained by STRING analysis is reported in Supplementary File S2; only proteins with significant strict FDR (<0.01) were considered and indicated with the gene name.

### 3.4. Preliminary Quantitative Comparison

Even though the AD and HC salivary samples were analyzed as pooled samples, we tentatively performed a quantitative comparative analysis of the triplicate IPs analyzed under R conditions. To obtain more robust statistical results, a quality check of the quantitative data was performed, as shown in Supplementary Figure S3. The total protein abundances calculated by PD software and their variances among the replicates of AD and HC CoIPs in R samples appeared comparable without (panel A, Figure S3) and with (panel B, Figure S3) the normalization of the total peptide amount. In the chart with normalized data (panel B), the variances appeared more equal among the AD and HC replicates with respect to the unnormalized data. Indeed, the normalized data were used for the statistics. This result ensured that any possible difference measurable between AD and HC CoIP was not attributable to uneven extraction of peptides from SDS-PAGE gels or to other experimental steps.

**Figure 5:**
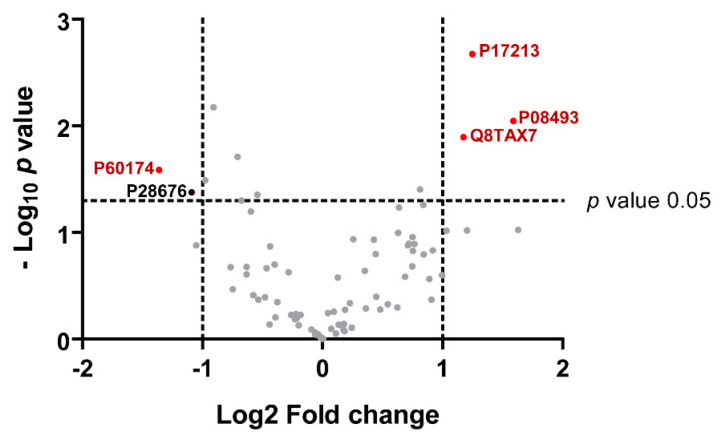
Volcano Plot of significantly increased (red dots on the right) or decreased (red dots on the left) proteins marked with their UniProt-KB codes in AD compared to HC group.

The results of the quantitative comparison of cystatin B and its 81 partner levels between AD and HC IPs are shown in Table 3 and in the volcano plot in Figure 5. The *p*-values and fold changes of the proteins with significant different abundances between AD and HC are indicated in Table 3. Triosephosphate isomerase (TPI) exhibited a lower level in the AD group, while significantly higher levels of bactericidal permeability-increasing protein (BPI), matrix γ-carboxyglutamic acid–rich (Gla) protein, and Mucin-7 (MUC-7) were found. A fifth protein, grancalcin, was individuated by a volcano plot with a slight abundance difference between AD and HC groups; a -Log10 *p* value of 1.4 (0.04) and a Log2 of fold change −1.1 (0.5) were calculated by the statistical analysis. Even if the fold change is good, the low significance of the *p* value made us consider the protein borderline.

## 4. Discussion

This explorative proteomic study, based on an AP-MS approach, was inspired by the results that we obtained in previous top-down proteomic investigations on the salivary proteome associated with Alzheimer’s disease. We previously demonstrated that some salivary proteins/peptides, including S-modified proteoforms of cystatin B, significantly varied in abundance between AD patients and healthy controls [14,15]. Due to the relevance of developing proteomic studies in peripheral biofluids to the AD-associated biomarker discovery and the known ability of cystatin B to interact in multi-protein complexes [24,25,26,27] and considering the significance of PPI studies in the field of neurodegenerative disease, we decided to investigate the possible PPI of cystatin B in AD saliva. The results obtained in this AP-MS based study showed that salivary cystatin B is involved in a multiprotein complex present in both AD and HC, samples and that we were able to immune-precipitate and characterize it for the first time in human saliva in vivo. Moreover, although preliminary, a quantitative comparison between the two groups showed interesting and significant differences for specific cystatin B interactors.

The results obtained in this explorative AP-MS study represent a starting point for next proteomic investigations, supported by technical verification with immunological techniques, to be performed on a larger cohort of patients at different stages of the disease progression and on other neurodegenerative diseases. It has been repeatedly stressed by various research groups that understanding the events responsible for AD pathogenesis requires the use of different proteomic strategies [17,18,20,42]; as suggested by Jain AP et al. the understanding of molecular mechanisms and cellular signaling pathways involved in AD pathogenesis is needed for discovering new targets and developing new therapeutic strategies [20], as well as for the diagnostic and disease progression monitoring. To date, several proteomic studies have identified candidate biomarkers of AD in brain tissue and CSF [17,18,20,42]. AD diagnosis is primarily based on clinical parameters, brain inspection, and CNS detection of Aβ and tau proteins that were shown to have excellent diagnostic accuracy when measured in CSF [17,42]. However, its invasive collection, the high heterogeneity of the patient population, and the complexity of AD pathogenesis highlight the need to find additional markers. Peripheral biomarkers may solve these limitations, especially by providing non-invasive solutions for disease diagnosis, and peripheral tissues and biofluids (blood cells, plasma, eye tissues, skin fibroblasts, urine, and saliva) show to be indicators of cognitive and biological changes in the brain and are supposed to differentiate neurological and normal conditions [19,20]. Studies on proteomic changes in blood plasma deserve special attention for neurodegenerative diseases such as AD and are of increasing interest due to the much less invasive method of sample collection as compared to CSF [17,18,20,43]. Saliva is more advantageous than blood for the much lower invasiveness and feasibility of the collection, which does not require healthcare personnel, and the good tolerability by the donors. It is a biofluid poorly studied in the AD field, and it shows the potentiality to provide very interesting insights on the AD pathogenesis and peripheral candidate biomarkers of the disease [14,15,20,28,30,31].

This study established a good and accurate approach to characterize PPIs in saliva and highlight significant variations in pathological conditions such as AD. Disease mechanisms are often mediated by protein complexes, and a PPI mapping approach may be useful to reveal disease mechanisms and therapeutic targets [44], especially for diseases such as AD, which is a complex pathology with a multitude of environmental and genetic factors contributing to its onset and progression. The ability of cystatin B to interact with other proteins was already shown by other investigations [25,26,27], as was its ability to form polymeric structures [24]. Non-covalent mechanisms of cystatin B in vitro oligomerization have been proposed as the model of domain-swapping for the dimeric form and the isomerization from *trans* to *cis* of a proline residue to form a cystatin B tetrameric structure [45]. Oligomers/polymers of cystatin B have never been observed in adult human saliva, while its different proteoforms derived from oxidation of the unique cysteine residue are typically detectable, as the glutathionylated, the cysteinylated, and the homodimer forms [40]. Interestingly, SDS-PAGE of IPs performed under non reducing conditions showed that cystatin B existed in different forms in the immunoprecipitated samples, as characterized in vivo by our AP-MS study. Indeed, cystatin B was characterized not only in the gel band at MW > 250 kDa, indicating its inclusion into the multiprotein complex, but also in the gel bands corresponding to the expected MW for the S-modified monomeric and dimeric proteoforms. Oligomeric/polymeric forms of cystatin B might be part of the salivary multiprotein complex, in accordance with the study of Cipollini et al. demonstrating that cystatin B assembles into polymeric structures that are SDS-resistant in vivo [24]. This is certainly an interesting hypothesis to evaluate in future investigations. The S-modified proteoforms might also be part of the immunoprecipitated complex, and we observed them in NR samples not only because they derive from the whole saliva but also because of their SDS-induced dissociation from the multiprotein complex.

Despite the interesting hypotheses that can be made, the methodological approach here applied did not allow obtaining insights on which cystatin B proteoform has been involved in the immunoprecipitated complex, if polymeric or monomeric, unmodified or S-modified. Indeed, the present AP-MS study was optimized to identify protein interactors forming with cystatin B, the multiprotein complex that we detected in saliva. Eighty-one cystatin B interactors were identified in samples analyzed under reducing conditions, and 113 interactors were identified under non reducing conditions. Theses interactors were commonly present in both AD and HC salivary pools. Fifty-four out of the 81 interactors identified in the R samples were also characterized in the samples obtained from the gel slices above 150 kDa in SDS-PAGE performed under the NR condition, showing that the interactions of a subset of proteins co-immunoprecipitated with cystatin B were resistant to SDS treatment, while others with weaker interactions were efficiently separated also in the absence of a reducing agent. Although more proteins were identified in NR samples than in R samples, we preferred to consider as accurately characterized cystatin B interactors those found in R samples, which were selected by excluding non-specific interactors based on both the CRAPome score and comparison with NEG samples, as described in the “Material and Methods” section.

The current study highlighted biological processes significantly represented in the salivary cystatin B interactome, such as exocytosis, granulocyte migration, neutrophil activation, antibacterial activity, modulation of the cytoskeleton and extra-cellular matrix, actin nucleation, peptidase inhibitor activity, and glucose. Moreover, it was highlighted that the interactors co-immunoprecipitated in the multiprotein complex cystatin B-linked are commonly found in plasma and blood and lymphatic cells, as leukocytes and platelets, and in the oral cavity, especially in salivary fluid, with some of the interactors specifically secreted by salivary glands.

The principal biological role of cystatin B is to interact with and inhibit cathepsins B, L, and H, the lysosomal cysteine proteases of the papain family [1]. None of them co-immunoprecipitated with cystatin B, according to the study of Di Giaimo and colleagues. Indeed, they found cystatin B to be part of a tissue-specific multiprotein complex in rat cerebellum, together with cytoplasmic proteins involved in the regulation of cytoskeletal functions but not with any protease [27]. They identified a multiprotein complex in rat cerebellum that showed several similarities with that identified in human saliva by our group, particularly with regard to proteins involved in the modulation of the cytoskeleton. Another study by this research group suggested that cystatin B may play a role in brain plasticity and that its deregulation could be involved in neurodegenerative and neuropsychiatric diseases [46]. According to these studies, several interactors identified in our study can remodel the actin cytoskeleton such as actin-related proteins [47], some of which are calcium-dependent proteins and are involved in the neutrophil activation processes. The reorganization of actin and cytoskeleton is required for granulocyte migration and neutrophil stimulation [48] and for degranulation and release of granule proteins to the extracellular milieu by the phagosome [49]. The translocation and exocytosis of granules by neutrophils require an increase of intracellular Ca^2+^ levels, mediated by numerous target proteins such as annexins and calmodulin [50]. However, we identified into the cystatin B interactome annexins A1, A3, A5, and A11, grancalcin, gelsolin, plastin-2, S100A6, which are calcium-binding proteins participating in the adhesion of neutrophils to fibronectin [51,52] and in the reorganization of actin cytoskeleton [53,54,55]. S100A8 and S100A9 were also found to participate in the salivary cystatin B interactome; they represent the main protein content of the neutrophils [56], where they are constitutively expressed as Ca^2+^ sensors, contributing to the cytoskeleton rearrangement and arachidonic acid metabolism [57]. In previous studies, we identified S100A8, S100A9, and their oxidized proteoforms as candidate salivary biomarkers of AD [14,15]. In addition, myeloperoxidase, CD63, elastase, cathepsin G, bactericidal permeability-increasing protein, CLD, and “peptides from neutrophil defensin 1 precursor” have been identified among the other proteins participating in the cystatin B interactome, which are typically expressed by neutrophils and are involved in innate immune-response [41,48].

Other cystatin B interactors found in this study were the integrin alpha-M/beta-2 complex and fibrinogen alpha, beta, and gamma chains. Integrin alpha-M/beta-2 complex can promote neutrophil adhesion ligand in vitro and can interact with both fibrinogen and complement systems [58]. Moreover, integrins can regulate the expression and activity of metalloproteinases that are involved in extracellular matrix remodeling [59]. Additionally, a cluster of proteins involved in metabolism were identified as cystatin B interactors, including 6-phosphogluconate dehydrogenase, glucose-6-phosphate dehydrogenase, glucose-6-phosphate isomerase, transketolase, and triosephosphate isomerase (TPI), enzymes involved in the pentose phosphate pathway and glycolysis. Moreover, several protease inhibitors have been identified as cystatin B interactors, such as alpha-1-antitrypsin and serpin B4, which are involved in the inflammatory response [60], cystatin C, D, SA, and SN, cathepsin inhibitors [1], and extracellular matrix protein 1, which inhibits matrix metalloproteinase-9 [61].

The quantitative data obtained in this study were considered preliminary due to the limitations related to the low volumes and/or low concentrations of several samples collected from AD patients, which required the use of pooled samples from the same group. Indeed, the strength of the quantitative comparison between proteins identified in IPs from salivary pools was certainly less than that achievable with IPs from single samples, although triplicate IP samples were used and quantitative data quality control preceded the statistical analysis. The quantitative comparison of cystatin B and its 81 partner levels between AD patients and HC IPs evidenced significant differences in certain proteins. Some components of the salivary multiprotein complex have already been characterized in our previous investigations on the salivary proteome of AD patients, such as S100A8, S100A9, and α-defensin 1, in addition to cystatin B. They had been found at significantly higher levels in the saliva of AD patients with respect to the healthy control group [14,15], while in the present study, non-significant variations were observed for these components. However, our previous studies have been performed using a top-down approach on the protein fraction soluble in acidic solution, while in the present study we analyzed, using a bottom-up approach, an immunoprecipitated multiprotein complex containing cystatin B interactors, in which the abundance of a protein does not necessarily reflect that present in the oral cavity. Moreover, we could not establish which proteoform/s of S100A8 and S100A9, as well as cystatin B, were included in the multiprotein complex and in what stoichiometric ratio with respect to other proteins and with respect to all the proteoforms of the parent protein. In the case of S100A8 and S100A9, although all the PTMs known for these proteins have been set in the PD data elaboration, only unmodified fragments were identified. In the case of cystatin B, the total protein immunoprecipitated from saliva was quantified and did not change between AD patients and healthy controls. Indeed, the IPs, analyzed by reducing SDS-PAGE and following western blotting, showed a unique positive signal of the protein that corresponded to the total unmodified monomeric cystatin B obtained in those experimental conditions.

Some cystatin B interactors showed different abundances between the two groups. TPI, which is a glycolytic enzyme, exhibited a lower abundance in the multiprotein complex immunoprecipitated from the patient group. Interestingly, TPI was associated with AD pathogenesis in other studies as well. In neuroblastoma cell cultures, it was demonstrated that TPI is particularly prone to nitrotyrosination induced by Aβ42 oligomers, a phenomenon causing a functional deficiency of the enzyme, which by generating the toxic by-product methylglyoxal leads to neuronal death [62,63]. Nitrosylated TPI was not detected in our IP samples, where the tryptic fragment carrying the nitro-tyrosine (at the 165 or 209 position, as suggested by Tajes and colleagues [62]) has not been revealed. However, the characterization of the oxidative modification of TPI involved in the cystatin B interactome occurring in the saliva of AD patients is worthy of further study, as is the characterization of other oxidative protein modifications. Indeed, oxidative stress conditions are a hallmark of AD pathogenesis. Neurons are particularly susceptible to the overproduction of reactive oxygen and nitrogen species (ROS/RNS), which is the most common consequence either of exposure to environmental risk factors or neurodegenerative processes. In these processes, the balance between the generation of ROS/RNS and the availability of cell defense systems may be dysfunctional [64,65,66]. In our previous studies of AD patient saliva [14,15], the nitrosylated form of the S100A8 protein was detectable in the patient group but not detectable in healthy controls. Several proteins prone to oxidation have been identified in AD and thus may contribute to a loss of normal cell function, especially numerous aberrant cysteine nitrosylated proteins, which have been detected in AD patient brains (from autopsy) or AD animal models [64,65,67]. Nitrosylated proteins such as protein disulfide isomerases, glyceraldehyde 3-phosphate, insulin degrading enzyme, and others have been observed in AD patients, suggesting a toxic modification and emphasizing the importance of better understanding the nytroso-proteins in the context of AD [67].

The cystatin B interactome of the AD group showed higher levels with respect to healthy controls of matrix Gla protein, BPI, and mucin-7. Matrix Gla protein is a Ca^2+^-binding and vitamin K-dependent protein that is synthesized in many mesenchymal cells, acting as a calcification-inhibitor and able to regulate the formation of matrix vesicles, the formation of apoptotic bodies, and the differentiation of vascular smooth muscle cells [68]. A possible role of cystatin B in the modulation of the extracellular environment has been recently proposed [11], which could explain the functional association with matrix gla protein in the salivary PPI highlighted in the present study.

Bactericidal permeability-increasing protein (BPI) is an antibacterial polypeptide that is cytotoxic to Gram-negative bacteria [69] and is released by neutrophils [48]. Mucin-7 is a small mucin involved in the antimicrobial humoral immune response of the oral cavity, where it participates in the clearance of bacteria [70]. The high abundance of antimicrobial proteins/peptides in the cystatin B interactome of AD patients, as observed here, is coherent with our previous findings in the acid soluble fraction of salivary proteins [14,15], where we observed significantly higher levels of antimicrobial proteins and peptides involved in the innate immune response. It is not surprising to find high levels of antimicrobial agents in the oral cavity of AD patients. Exposure to bacterial infections is considered one of the risk factors in AD pathogenesis. It has been hypothesized that oral and gut microbiota may alter the permeability of the blood-brain barrier, facilitating the colonization by opportunistic pathogens and inducing microglia activation and a neuroinflammatory response leading to neuronal loss and neurodegeneration by favoring Aβ accumulation, tau hyperphosphorylation, and the disintegration of neurotransmitters into toxic metabolites [71].

## 5. Conclusions

To our knowledge, this is the first comprehensive study on the cystatin B interactome in saliva. Our study revealed that salivary cystatin B undergoes PPIs, with several proteins participating in specific biological functions, such as degranulation of granulocytes, activation of neutrophils, cytoskeleton modulation, anti-microbial defense, and glucose metabolism. Moreover, the cystatin B interactome appeared similar between patients and controls except for the abundance of specific proteins, some of which are of great interest for AD pathogenesis. The preliminary quantitative comparison suggested that the lower level of TPI in AD patients might be further investigated as a potential AD peripheral biomarker in a larger AD sample. Moreover, TPI in the AD group should be further investigated, especially for its nitro-tyrosine proteoform, stimulating future investigations by using the salivary biofluid as a source of information on the implications of oxidative changes at proteomic levels in AD pathogenesis.

Another interesting feature we highlighted was the detection of significantly different levels of anti-microbial proteins in the cystatin B interactome from AD patients and healthy controls, a result that reinforces the hypothesis that AD is a disease associated with both infections and the innate immune response.

## Figures and Tables

**Table 1:** Demographic data of the subjects included in the three groups: HC, AD and NEG. For each subject, total protein concentration expressed in μg/μL of whole saliva was reported.

HC	Sex, Age	μg/μL	AD	Sex, Age	μg/μL	NEG	Sex, Age	μg/μL
#3	F, 84	1.1	#12	F, 84	0.6	#2	M, 85	1.8
#5	F, 81	1.4	#13	F, 79	0.7	#3	F, 84	1.1
#9	M, 71	1.5	#14	F, 82	0.9	#4	M, 82	1.6
#11	M, 76	1.7	#16	F, 83	0.6	#5	F, 81	1.4
#12	F, 77	1.2	#17	F, 63	0.7	#7	F, 79	1.6
#13	M, 74	1.2	#18	F, 80	0.9	#8	M, 74	2.2
#14	M, 87	2.7	#19	F, 80	0.4	#9	M, 71	1.5
#16	F, 81	0.9	#20	M, 87	0.5	#10	M, 78	1.5
#17	F, 82	1.5	#22	M, 87	0.8	#11	M, 76	1.7
#19	F, 86	1.8	#23	F, 75	0.4	#12	F, 77	1.2
#20	F, 73	3.2	#24	F, 75	0.3	#13	M, 74	1.2
#21	M, 79	2.2	#25	F, 83	0.2	#15	M, 73	1.7
#22	F, 78	1.7	#26	F, 84	0.4	#16	F, 81	0.9
#24	F, 78	1.2	#27	F, 81	0.3	#18	F, 72	1.7
#25	F, 75	2.1	#30	M, 86	0.2	#20	F, 73	3.2
#26	F, 75	0.8	#32	F, 88	0.3	#21	M, 79	2.2
#30	F, 76	0.4	#33	F, 81	0.3	#22	F, 78	1.7
#28	F, 78	0.5	#34	F, 77	0.4	#23	F, 79	2.1
#31	F, 81	0.4	#35	M, 87	0.8	#24	F, 78	1.2
#34	F, 72	0.2	#36	M, 84	1.0	#25	F, 75	2.1
#35	M, 80	1.2	#37	F, 77	0.6	#26	F, 75	0.8
#37	F, 78	3.6	#38	F, 78	1.2	#28	F, 78	0.5
#38	F, 78	2.2	#39	M, 76	0.8	#29	M, 73	0.3
#39	F, 83	1.7	#40	M, 85	1.6	#35	M, 80	1.2
						#36	F, 73	2.9
						#38	F, 78	2.2

**Table 2:** Proteins identified as cystatin B interactors in R samples in both AD and HC groups. For each protein the Uniprot-KB code and the molecular weight (MW) are reported. The MW of the precursor pro-protein is indicated for the small peptides when the sequence identified by the PD software could not be attributed to a certain peptide. The dot ● indicates proteins identified also in the gel-slices above 150 kDa in NR samples of both groups.

UniProt-KB Code	Protein Name	MW	AD and HC (NR Samples) > 150 kDa
P52209	6-phosphogluconate dehydrogenase, decarboxylating	53.1	●
O15144	Actin-related protein 2/3 complex subunit 2	34.3	●
P59998	Actin-related protein 2/3 complex subunit 4	19.7	
P61158	Actin-related protein 3	47.3	●
Q9HDC9	Adipocyte plasma membrane-associated protein	46.5	
P01009	Alpha-1-antitrypsin	46.7	
A8K2U0	Alpha-2-macroglobulin-like protein 1	161.0	●
P0DTE7	Alpha-amylase 1B	57.7	●
P04083	Annexin A1	38.7	●
P50995	Annexin A11	54.4	
P12429	Annexin A3	36.4	
P08758	Annexin A5	35.9	
P20292	Arachidonate 5-lipoxygenase-activating protein	18.1	
P17213	Bactericidal permeability-increasing protein	53.9	●
Q96DR5	BPI fold-containing family A member 2	27.0	
Q8TDL5	BPI fold-containing family B member 1	52.4	●
Q8N4F0	BPI fold-containing family B member 2	49.1	
P23280	Carbonic anhydrase 6	35.3	●
P06731	Carcinoembryonic antigen-related cell adhesion molecule 5	76.7	
P49913	Cathelin-like domain	11.3	
P08311	Cathepsin G	28.8	●
P08962	CD63 antigen	25.6	
P29373	Cellular retinoic acid-binding protein 2	15.7	●
P0C0L4	Complement C4-A	192.7	
O75131	Copine-3	60.1	
Q9UBG3	Cornulin	53.5	●
P31146	Coronin-1A	51.0	●
P04080	Cystatin-B	11.1	●
P01034	Cystatin-C	15.8	●
P28325	Cystatin-D	16.1	●
P09228	Cystatin-SA	16.4	
P01037	Cystatin-SN	16.4	●
P54108	Cysteine-rich secretory protein 3	27.6	
P31930	Cytochrome b-c1 complex subunit 1	52.6	
P32926	Desmoglein-3	107.5	●
Q96HE7	ERO1-like protein alpha	54.4	●
Q16610	Extracellular matrix protein 1	60.6	●
Q01469	Fatty acid-binding protein 5	15.2	●
P02671	Fibrinogen alpha chain	94.9	●
P02675	Fibrinogen beta chain	55.9	●
P02679	Fibrinogen gamma chain	51.5	●
P17931	Galectin-3	26.1	●
P47929	Galectin-7	15.1	●
P06396	Gelsolin	85.6	●
P11413	Glucose-6-phosphate 1-dehydrogenase	59.2	●
P06744	Glucose-6-phosphate isomerase	63.1	●
P15104	Glutamine synthetase	42.0	
P09211	Glutathione S-transferase P	23.3	●
P28676	Grancalcin	24.0	
P04899	Guanine nucleotide-binding protein G(i) subunit alpha-2	40.4	●
P04792	Heat shock protein beta-1	22.8	●
P11215	Integrin alpha-M	127.1	
P05107	Integrin beta-2	84.7	
P22079	Lactoperoxidase	80.2	●
P30740	Leukocyte elastase inhibitor	42.7	●
P08493	Matrix γ-carboxyglutamic acid (GLA)–rich protein	12.3	●
P14780	Matrix metalloproteinase-9	78.4	
Q9HC84	Mucin-5B	596.0	●
Q8TAX7	Mucin-7	39.1	●
P24158	Myeloblastin	27.8	
P41218	Myeloid cell nuclear differentiation antigen	45.8	●
P05164	Myeloperoxidase	83.8	●
P59665	Neutrophil defensin 1	10.2	●
P08246	Neutrophil elastase	28.5	●
O15162	Phospholipid scramblase 1	35.0	
P13796	Plastin-2	70.2	●
P12273	Prolactin-inducible protein	16.6	●
P06703	Protein S100-A6	10.2	●
P05109	Protein S100-A8	10.8	●
P06702	Protein S100-A9	13.2	●
Q08188	Protein-glutamine gamma-glutamyltransferase E	76.6	●
P50395	Rab GDP dissociation inhibitor beta	50.6	●
P60763	Ras-related C3 botulinum toxin substrate 3	21.4	
P51159	Ras-related protein Rab-27A	24.9	
P52566	Rho GDP-dissociation inhibitor 2	23.0	●
P48594	Serpin B4	44.8	
P36952	Serpin B5	42.1	●
Q9UBC9	Small proline-rich protein 3	18.1	●
P29401	Transketolase	67.8	●
P60174	Triosephosphate isomerase	26.7	●
Q16851	UTP--glucose-1-phosphate uridylyltransferase	56.9	
Q96DA0	Zymogen granule protein 16 homolog B	22.7	●

**Table 3:** Proteins with significant different abundances between AD and HC groups. For each protein the UniProt-KB code, the LFQ abundance in Log2 scale expressed as mean of each replicate ± standard deviation (SD), the *p* value and the fold change calculated by Perseus are indicated with and without logarithmic form, −Log10 and Log2 respectively. BPI, Bactericidal permeability-increasing protein; Matrix Gla protein, matrix γ-carboxyglutamic acid–rich protein; TPI, Trioso-phosphate isomerase. Significant increased, or decreased, protein abbundance in AD is symbolized by ↑ or ↓, respectively.

		Log2 LFQ Abundance, Mean ± SD		AD VS HC		
UniProt-KB Code	Protein Name	AD	HC	−Log10 *p* Value (*p* Value)	Log2 Fold Change (Fold Change)	
P17213	BPI	24.8 ± 0.1	23.5 ± 0.3	2.7 (0.002)	1.2 (2.3)	↑AD
P08493	Matrix Gla protein	22.2 ± 0.4	20.6 ± 0.4	2.1 (0.008)	1.6 (3.0)	↑AD
Q8TAX7	Mucin-7	24.7 ± 0.5	23.6 ± 0.2	1.9 (0.01)	1.2 (2.3)	↑AD
P60174	TPI	18.3 ± 0.6	19.7 ± 0.4	1.6 (0.02)	−1.4 (0.4)	↓AD

## Data Availability

HR-MS/MS data are freely available at the ProteomeXchange Consortium (http://ww.ebi.ac.uk/pride (accessed on 11 January 2023)) with the dataset identifiers PXD030679 and PXD039286. PXD030679: Username: only for reviewer_pxd030679@ebi.ac.uk; Password: GbsSp1TU; PXD039286: Username: only for reviewer_pxd039286@ebi.ac.uk; Password: bTxT0kae.

## Supplementary Materials

The following are available online at https://www.mdpi.com/article/10.3390/life13030748/s1, Supplementary File S1. Table S1: Pharmacological treatment and clinical features of AD patients. Table S2: total protein concentration measured in each sample, and volume utilized to constitute the three different pools. Table S3: Proteins identified with high confidence under R and NR conditions for AD and HC groups, and under R conditions for NEG samples. For each protein, the UniProt-KB code is indicated, together with the number of unique peptides identified, the percentage of coverage, the SEQUEST-HT score obtained by PD software, and the observed PTMs; Table S4: Results of the student t-test for the comparison of the protein LFQ abundances among the AD, HC, and NEG groups. Proteins marked in grey were excluded as unspecific interactors according to one or both of the following conditions: i) higher (↑) or unchanged (ns) LFQ levels in AD and HC groups with respect to NEG (R samples only); ii) a CRAPome score of the found/total ratio over the 25% of total experiments deposited in the database (R and NR samples). For each protein in the Uniprot-KB code, the LFQ abundance in log2 scale is expressed as the mean of each replicate ± standard deviation (SD), the *p* value, and the fold change calculated by Perseus (in R samples) are reported; Table S5: Qualitative comparison of the proteins identified in CoIPs from AD and HC analyzed under R and NR conditions, after the application of the exclusion criteria. For each protein, the Uniprot-KB code is reported, and the symbol ● indicates whether the protein was identified under R, NR, or both conditions; Figure S1: SDS-PAGE under NR and R conditions of IP proteins after cystatin B Co-IP assay from AD and HC pools in triplicate (A) and SDS-PAGE under R condition of IP proteins after normal mouse Co-IP assay from NEG pool in duplicate (B). Black lines indicate how each lane has been cut prior to tryptic digestion and nano-HPLC-HR-ESI-MS/MS analysis; Figure S2: Volcano Plots of significantly enriched proteins in AD (blue dots) with respect to NEG (A) and significantly enriched proteins in HC (green dots) with respect to NEG (B). In both panels, red dots represent enriched proteins in NEG, while grey dots represent unchanged proteins; Figure S3: Chart of comparison of the total protein LFQ abundances (Log10) determined by PD software in AD and HC R samples (triplicates) without (panel A) and with (panel B) normalization with respect to the total peptide amount measured in the same samples. The central line represents the variance related to each replicate. SD deviation is indicated. The protein outliers are indicated with black squares. Supplementary file S2. Detailed information is obtained by STRING analysis.
