# Supplementary material for: Characterization of Cystatin B Interactome in Saliva from Healthy Elderly and Alzheimer’s Disease Patients
Source: Life (Basel). 2023 Mar 10;13(3):748. doi: 10.3390/life13030748 (PMC10054399; doi:10.3390/life13030748)

SUPPLEMENTARY MATERIAL

Table S1: Pharmacological treatment and clinical features of AD patients.

AD	Sex, age	AD Diagnosis	AD Therapy
#12	F,84	Moderate AD	Rivastigmine
#13	F,79	Mild AD	Rivastigmine
#14	F,82	Mild AD	Donezepil
#16	F,83	Moderate AD	Donezepil + Memantine
#17	F,63	Mild AD	Donezepil
#18	F,80	Mild AD	Rivastigmine
#19	F,80	Mild AD	Rivastigmine
#20	M,87	Mild AD	Memantine
#22	M,87	Mild AD	Donezepil
#23	F,75	Mild AD	Rivastigmine
#24	F,75	Mild AD	Rivastigmine
#25	F,83	Mild AD	Rivastigmine
#26	F,84	Moderate AD	Rivastigmine
#27	F,81	Mild AD	Rivastigmine
#30	M,86	Moderate AD	Memantine
#32	F,88	Moderate AD	Memantine
#33	F,81	Moderate AD	Memantine
#34	F,77	Moderate AD	Rivastigmine + Memantine
#35	M,87	Mild AD	Memantine
#36	M,84	Mild AD	Memantine
#37	F,77	Mild AD	Rivastigmine
#38	F,78	Mild AD	Rivastigmine
#39	M,76	Moderate AD	Rivastigmine + Memantine
#40	M,85	Mild AD	Memantine

Table S2: Total protein concentration measured in each sample, and volume utilized to constitute the three different pools.

	µg/µl	µg	µl		µg/µl	µg	µl		µg/µl	µg	µl
pool	0.95	1600	1688	pool	0.46	1600	3442	pool	1.23	800	650
aliquot	0.95	400	422	aliquot	0.46	400.02	860	aliquot	1.23	400	325
**HC**	**µg/µl**	**µg**	**µl**	**AD**	**µg/µl**	**µg**	**µl**	**NEG**	**µg/µl**	**µg**	**µl**
**#3**	1.1	66.67	60.6	**#12**	0.6	66.67	111.1	**#2**	1.8	30.77	17.1
**#5**	1.4	66.67	47.6	**#13**	0.7	66.67	95.2	**#3**	1.1	30.77	28.0
**#9**	1.5	66.67	44.4	**#14**	0.9	66.67	74.1	**#4**	1.6	30.77	19.2
**#11**	1.7	66.67	39.2	**#16**	0.6	66.67	111.1	**#5**	1.4	30.77	22.0
**#12**	1.2	66.67	55.6	**#17**	0.7	66.67	95.2	**#7**	1.6	30.77	19.2
**#13**	1.2	66.67	55.6	**#18**	0.9	66.67	74.1	**#8**	2.2	30.77	14.0
**#14**	2.7	66.67	24.7	**#19**	0.4	66.67	166.7	**#9**	1.5	30.77	20.5
**#16**	0.9	66.67	74.1	**#20**	0.5	66.67	133.3	**#10**	1.5	30.77	20.5
**#17**	1.5	66.67	44.4	**#22**	0.8	66.67	83.3	**#11**	1.7	30.77	18.1
**#19**	1.8	66.67	37.0	**#23**	0.4	66.67	166.7	**#12**	1.2	30.77	25.6
**#20**	3.2	66.67	20.8	**#24**	0.3	66.67	222.2	**#13**	1.2	30.77	25.6
**#21**	2.2	66.67	30.3	**#25**	0.2	66.67	333.4	**#15**	1.7	30.77	18.1

**#22**	1.7	66.67	39.2	**#26**	0.4	66.67	166.7	**#16**	0.9	30.77	34.2
**#24**	1.2	66.67	55.6	**#27**	0.3	66.67	222.2	**#18**	1.7	30.77	18.1
**#25**	2.1	66.67	31.7	**#30**	0.2	66.67	333.4	**#20**	3.2	30.77	9.6
**#26**	0.8	66.67	83.3	**#32**	0.3	66.67	222.2	**#21**	2.2	30.77	14.0
**#30**	0.4	66.67	166.7	**#33**	0.3	66.67	222.2	**#22**	1.7	30.77	18.1
**#28**	0.5	66.67	133.3	**#34**	0.4	66.67	166.7	**#23**	2.1	30.77	14.7
**#31**	0.4	66.67	166.7	**#35**	0.8	66.67	83.3	**#24**	1.2	30.77	25.6
**#34**	0.2	66.67	333.4	**#36**	1	66.67	66.7	**#25**	2.1	30.77	14.7
**#35**	1.2	66.67	55.6	**#37**	0.6	66.67	111.1	**#26**	0.8	30.77	38.5
**#37**	3.6	66.67	18.5	**#38**	1.2	66.67	55.6	**#28**	0.5	30.77	61.5
**#38**	2.2	66.67	30.3	**#39**	0.8	66.67	83.3	**#29**	0.3	30.77	102.6
**#39**	1.7	66.67	39.2	**#40**	1.6	66.67	41.7	**#35**	1.2	30.77	25.6
								**#36**	2.9	30.77	10.6
								**#38**	2.2	30.77	14.0

Table S3: Proteins identified with high confidence under R and NR conditions for AD and HC groups, and under R conditions for NEG samples. For each protein, the UniProt-KB code is indicated, together with the number of unique peptides identified, the percentage of coverage and the SEQUEST-HT score obtained by PD software, and the observed PTMs.

UniProt-KB Code	Protein name	AD and HC (R)				AD and HC (NR)				NEG (R)		
		Cov. %	Unique Peptides	Score	PTM	Cov. %	Unique Peptides	Score	PTM	Cov. %	Unique Peptides	Score
P31946	14-3-3 protein beta/alpha	17	2	31.83		17	2	22.34				
P61981	14-3-3 protein gamma	17	2	33.64								
P31947	14-3-3 protein sigma	21	4	38.99		26	5	31.1				
P63104	14-3-3 protein zeta/delta	32	5	73.07		19	4	50.95				
P08865	40S ribosomal protein SA	7	2	5.59								
P52209	6-phosphogluconate dehydrogenase, decarboxylating	22	10	112.48		22	10	103.59		10	4	17.95
P62906	60S ribosomal protein L10a	10	2	0								
P30050	60S ribosomal protein L12									15	2	0
P60709	Actin, cytoplasmic 1									48	10	75.19
O15144	Actin-related protein 2/3 complex subunit 2	8	2	0		6	2	1.73				
P59998	Actin-related protein 2/3 complex subunit 4	11	2	1.76		16	3	2.19				
P61158	Actin-related protein 3	6	3	1.61		7	3	3.65				
P23526	Adenosylhomocysteinase	5	2	3.48		6	2	3.47				
Q01518	Adenylyl cyclase-associated protein 1	4	2	3.34		9	4	2.22				
Q9HDC9	Adipocyte plasma membrane-associated protein	12	5	1.82		9	4	0				
P61204	ADP-ribosylation factor 3	12	2	1.73								
P05141	ADP/ATP translocase 2	14	4	3.35	M_1-loss + NTA*							
P02768	Albumin	36	24	286.2		33	22	154.11		16	9	31.04
P12814	Alpha-actinin-1					9	2	15.72		3	2	1.99

UniProt-KB Code	Protein name	AD and HC (R)				AD and HC (NR)				NEG (R)		
		Cov. %	Unique Peptides	Score	PTM	Cov. %	Unique Peptides	Score	PTM	Cov. %	Unique Peptides	Score
P01009	Alpha-1-antitrypsin	7	3	0		9	3	1.87				
A8K2U0	Alpha-2-macroglobulin-like protein 1	4	6	8.62		7	9	12.88		2	3	1.68
P0DTE7	Alpha-amylase 1B	52	23	387.65		50	22	250.51		37	14	36.95
P06733	Alpha-enolase	12	5	5.24		21	7	14.15		26	5	11.93
P04083	Annexin A1	62	23	451.45	M_1-loss + NTA	60	23	412.81	M_1-loss + NTA	37	12	39.99
P50995	Annexin A11	4	2	0		6	3	8.76				
P07355	Annexin A2	47	15	116.87	M_1-loss + NTA	42	13	82.61	M_1-loss + NTA	14	5	11.38
P12429	Annexin A3	45	15	56.56	M_1-loss + NTA	44	14	34.24	M_1-loss + NTA	13	4	3.98
P08758	Annexin A5	5	2	3.36								
P03973	Antileukoproteinase	46	6	66.53		28	6	39.39		30	6	22.42
P02647	Apolipoprotein A-I	8	2	2.11						19	5	13.04
P20292	Arachidonate 5-lipoxygenase-activating protein	17	3	40.46		5	1	45.55				
P05089	Arginase-1					10	3	3.72		7	2	2.26
P25705	ATP synthase subunit alpha, mitochondrial	12	6	15.29		3	2	3.94				
P06576	ATP synthase subunit beta, mitochondrial	15	7	20.86		5	2	7.73				
P20160	Azurocidin	27	5	155.35		35	5	65.78		40	6	65.29
P17213	Bactericidal permeability-increasing protein	23	10	51.29		25	10	24.85		9	4	21.35
Q9NP55	BPI fold-containing family A member 1									13	2	0
Q96DR5	BPI fold-containing family A member 2	33	10	176.58		30	9	140.88		22	6	17.62
Q8TDL5	BPI fold-containing family B member 1	25	11	87.19		24	10	75.31		29	10	25.15

UniProt-KB Code	Protein name	AD and HC (R)				AD and HC (NR)				NEG (R)		
		Cov. %	Unique Peptides	Score	PTM	Cov. %	Unique Peptides	Score	PTM	Cov. %	Unique Peptides	Score
Q8N4F0	BPI fold-containing family B member 2	24	9	51.01		18	8	31.98		22	7	22.99
P12830	Cadherin-1	2	2	5.61		2	2	5.31		2	2	3.47
Q14CN2	Calcium-activated chloride channel regulator 4	2	2	0								
Q9NZT1	Calmodulin-like protein 5					48	5	8.86	M_1-loss + NTA	45	4	0
P23280	Carbonic anhydrase 6	20	5	90.41		20	5	80.13		14	3	17.3
P06731	Carcinoembryonic antigen-related cell adhesion molecule 5	3	2	3.77						3	2	1.78
P04040	Catalase					4	2	4.33		11	5	5.48
P49913	Cathelin-like domain	14	2	1.67		14	2	15.27				
P08311	Cathepsin G	45	14	454.14		43	11	317.88		39	9	63.69
P08962	CD63 antigen	5	2	0		4	1	1.83				
P29373	Cellular retinoic acid-binding protein 2	40	5	3.64		25	4	3.43	M_1-loss			
P23528	Cofilin-1	21	3	38.98	M_1-loss + NTA	26	4	33.85	M_1-loss + NTA			
P02747	Complement C1q subcomponent subunit C	21	4	22.74		10	2	1.6		23	4	16.1
P01024	Complement C3	2	3	0		5	9	3.49		4	5	1.76
P0C0L4	Complement C4-A	2	3	7.36		2	2	0				
O75131	Copine-3	3	2	0								
Q9UBG3	Cornulin	9	4	1.81		14	5	6.12				
P31146	Coronin-1A	12	6	19.13		20	9	25.63				
P04080	Cystatin-B	70	4	176.99	NTA	70	4	216.9	NTA	24	2	5.1
P01034	Cystatin-C	19	2	11.92		19	2	3.32		19	2	3.75
P28325	Cystatin-D	25	3	24.4		25	3	11.01				
P09228	Cystatin-SA	38	3	19.14		32	2	18.92				
P01037	Cystatin-SN	57	4	152.58		46	3	86.76		44	4	2.11

UniProt-KB Code	Protein name	AD and HC (R)				AD and HC (NR)				NEG (R)		
		Cov. %	Unique Peptides	Score	PTM	Cov. %	Unique Peptides	Score	PTM	Cov. %	Unique Peptides	Score
P54108	Cysteine-rich secretory protein 3	13	3	10.99		13	3	10				
P31930	Cytochrome b-c1 complex subunit 1	5	2	7.64								
Q9UGM3	Deleted in malignant brain tumors 1 protein	37	24	343.6		35	22	559.17		32	16	92.58
P81605	Dermcidin	23	3	25.7		23	3	31.84		13	2	8.36
Q08554	Desmocollin-1					9	6	12.79		5	4	8.99
Q02413	Desmoglein-1	2	2	1.74		19	13	13.38		14	9	6.74
P32926	Desmoglein-3	4	3	0		4	3	3.32		2	2	2.15
P15924	Desmoplakin	4	12	26.23		18	48	83.33		10	27	36.03
P68104	Elongation factor 1-alpha 1	13	6	42.19		15	7	46.37		21	4	13.87
P26641	Elongation factor 1-gamma	5	2	0		8	3	2.22				
P13639	Elongation factor 2					6	5	3.58		4	2	0
P12724	Eosinophil cationic protein	24	4	25.99		25	4	11.46		18	2	2.31
Q96HE7	ERO1-like protein alpha	7	4	0		12	6	2.28				
Q16610	Extracellular matrix protein 1	7	3	0		16	7	4.48				
P15311	Ezrin	11	4	10.4		12	4	10.64				
P52907	F-actin-capping protein subunit alpha-1	14	3	0	M_1-loss + NTA							
P47756	F-actin-capping protein subunit beta	9	2	3.5		12	3	1.6				
Q01469	Fatty acid-binding protein 5	44	5	32.23	M_1-loss + NTA	76	8	70.61	M_1-loss + NTA	25	3	2.65
P02671	Fibrinogen alpha chain	2	2	7.28		5	4	5.68				
P02675	Fibrinogen beta chain	20	7	17.09		23	8	9.22				
P02679	Fibrinogen gamma chain	14	6	43.41		17	8	52.03		11	4	9.14
Q5D862	Filaggrin-2					2	3	0		1	2	0
P04075	Fructose-bisphosphate aldolase A	24	8	37.44		28	9	64.96		15	3	2.2

UniProt-KB Code	Protein name	AD and HC (R)				AD and HC (NR)				NEG (R)		
		Cov. %	Unique Peptides	Score	PTM	Cov. %	Unique Peptides	Score	PTM	Cov. %	Unique Peptides	Score
P17931	Galectin-3	12	3	0		19	4	0				
Q08380	Galectin-3-binding protein	7	4	4.2		14	8	12.08		10	5	1.76
P47929	Galectin-7	14	2	10.15		27	3	10.53				
P06396	Gelsolin	4	3	14.89		6	5	0				
P11413	Glucose-6-phosphate 1-dehydrogenase	6	3	11.57	M_1-loss + NTA	19	10	1.89	M_1-loss + NTA			
P06744	Glucose-6-phosphate isomerase	14	7	0		15	7	8.1				
P15104	Glutamine synthetase	4	2	0								
P09211	Glutathione S-transferase P	36	6	21.01	M_1-loss	27	5	24.22	M_1-loss	34	4	9.35
P04406	Glyceraldehyde-3-phosphate dehydrogenase	36	10	56.83		42	11	63.44		21	5	7.46
Q9H4G4	Golgi-associated plant pathogenesis-related protein 1									17	2	4.02
P28676	Grancalcin	31	7	50.86		13	4	3.67		15	3	8.79
P04899	Guanine nucleotide-binding protein G(i) subunit alpha-2	7	2	3.7		10	3	1.62				
P00738	Haptoglobin	8	3	1.96						4	2	4.25
P0DMV9	Heat shock 70 kDa protein 1B	8	2	7.19		16	6	8.65		16	6	8.71
P04792	Heat shock protein beta-1	41	7	59.2	Phospho-S_{82}	34	5	11.24		13	2	4.39
P16402	Histone H1.3	16	4	12.88		22	5	18.98		7	2	2.21
P16401	Histone H1.5	12	3	11.85		18	4	18.4		12	3	0
Q16777	Histone H2A type 2-C									24	3	16.96
Q16778	Histone H2B type 2-E	48	2	115.6		46	2	137.17				
Q5QNW6	Histone H2B type 2-F	48	2	151.49		46	2	166.36				
P84243	Histone H3.3	20	5	176.09		29	6	179.51				
P62805	Histone H4	55	7	282.4		56	8	231.75		46	6	37.45
Q86YZ3	Hornerin	4	4	2.46		12	9	17.14		5	3	0
P11215	Integrin alpha-M	10	10	14.92		30	26	75.34		2	2	0
P05107	Integrin beta-2	11	8	16.94		10	7	25.83				

UniProt-KB Code	Protein name	AD and HC (R)				AD and HC (NR)				NEG (R)		
		Cov. %	Unique Peptides	Score	PTM	Cov. %	Unique Peptides	Score	PTM	Cov. %	Unique Peptides	Score
P14923	Junction plakoglobin	12	8	8.84		34	21	46.05		23	12	35.41
Q9P0G3	Kallikrein-14	21	4	1.78						18	3	4.74
P00338	L-lactate dehydrogenase A chain	22	7	82.54		19	5	90.53		18	4	11.25
P22079	Lactoperoxidase	3	2	9		10	7	19.5				
P02788	Lactotransferrin	62	43	527.47		62	42	546.49		64	42	179.9
P30740	Leukocyte elastase inhibitor	29	9	64.5		37	14	62.42		6	2	4.59
P61626	Lysozyme C	55	7	90.74		57	7	56.43		35	5	55.03
P08493	Matrix γ-carboxyglutamic acid (GLA)–rich protein	23	2	17.42		23	2	23.73				
P14780	Matrix metalloproteinase-9	3	2	1.68								
P26038	Moesin	8	2	4.98						9	2	5.93
Q9HC84	Mucin-5B	8	24	79.86		14	54	213.87		2	7	7.51
Q8TAX7	Mucin-7	4	2	36.83		7	3	34.53				
P24158	Myeloblastin	11	3	50.35		11	3	23.93		11	3	13.51
P41218	Myeloid cell nuclear differentiation antigen	20	8	6.72		10	5	10.04				
P05164	Myeloperoxidase	35	27	364.64		29	20	213.91		31	20	61.89
O14950	Myosin regulatory light chain 12B	24	4	1.68		18	3	5.41				
P35579	Myosin-9	16	29	33.72		26	52	105.48				
P59665	Neutrophil defensin 1	20	4	450.59		20	4	482.05		20	4	80.3
P08246	Neutrophil elastase	38	8	334.31		48	9	271.32		46	11	45.45
P23284	Peptidyl-prolyl cis-trans isomerase B									10	2	2.05
O75594	Peptidoglycan recognition protein 1	12	2	1.66						16	2	14.28
Q06830	Peroxiredoxin-1	35	5	89.73		44	7	98.06				
P32119	Peroxiredoxin-2	19	3	48.55		25	6	40.63				
Q13162	Peroxiredoxin-4	13	2	4.97		12	2	8.19				

UniProt-KB Code	Protein name	AD and HC (R)				AD and HC (NR)				NEG (R)		
		Cov. %	Unique Peptides	Score	PTM	Cov. %	Unique Peptides	Score	PTM	Cov. %	Unique Peptides	Score
P30041	Peroxiredoxin-6	17	4	1.68		28	6	10.87				
P00558	Phosphoglycerate kinase 1	8	3	6.94		18	6	6.41				
Q6P4A8	Phospholipase B-like 1									4	2	3.53
O15162	Phospholipid scramblase 1	7	2	0								
Q13835	Plakophilin-1	5	3	1.76		7	5	6.79		5	3	1.99
P13796	Plastin-2	15	5	37.95		25	12	51.01		4	2	3.95
P01833	Polymeric immunoglobulin receptor	42	30	371.92		31	27	406.05		43	28	147.67
P07737	Profilin-1	50	5	16.34		59	6	17.14				
P28799	Progranulin									7	3	5.54
P35232	Prohibitin	8	2	3.46								
P12273	Prolactin-inducible protein	66	9	115.27		49	8	84.48		53	7	31.06
P60900	Proteasome subunit alpha type-6	9	2	0								
P07237	Protein disulfide-isomerase	4	2	0		4	2	0				
P06703	Protein S100-A6	17	2	16.04		17	2	12.51				
P05109	Protein S100-A8	48	6	180.79		51	8	235.77		33	5	52.9
P06702	Protein S100-A9	56	8	152.53		53	7	155.55		48	5	2.04
Q08188	Protein-glutamine gamma-glutamyltransferase E	44	26	205.88	M_1-loss + NTA	58	34	264.38	M_1-loss + NTA	19	10	31.08
P22735	Protein-glutamine gamma-glutamyltransferase K					6	5	1.75		3	2	1.79
P14618	Pyruvate kinase PKM	17	9	59.02		29	13	104.29		10	4	8.48
P50395	Rab GDP dissociation inhibitor beta	4	2	0		12	6	0				
P60763	Ras-related C3 botulinum toxin substrate 3	15	3	5.36								
P51159	Ras-related protein Rab-27A	25	5	5.19	M_1-loss + NTA							
P52566	Rho GDP-dissociation inhibitor 2	10	2	0		10	2	3.76				

UniProt-KB Code	Protein name	AD and HC (R)				AD and HC (NR)				NEG (R)		
		Cov. %	Unique Peptides	Score	PTM	Cov. %	Unique Peptides	Score	PTM	Cov. %	Unique Peptides	Score
Q96P63	Serpin B12	4	2	1.69		11	4	0		14	4	7.34
P48594	Serpin B4	4	2	1.69								
P36952	Serpin B5	7	3	1.65		21	8	10.99				
Q9UBC9	Small proline-rich protein 3	38	4	19.89		47	7	23.7				
P29401	Transketolase	4	3	5.13		12	8	9.33				
P60174	Triosephosphate isomerase	10	2	6.28		19	4	4.34		11	2	2.33
P0DPH8	Tubulin alpha-3D chain	5	2	10.56								
Q16851	UTP--glucose-1-phosphate uridylyltransferase	4	2	1.68		2	1	0				
P08670	Vimentin	5	2	1.71		4	2	3.43				
P21796	Voltage-dependent anion-selective channel protein 1	8	2	3.95	M_1-loss + NTA							
P45880	Voltage-dependent anion-selective channel protein 2	7	2	2.03								
Q96DA0	Zymogen granule protein 16 homolog B	53	9	276.09		49	8	231.68		45	6	44.54
Proteins identified only in NR samples												
P61160	Actin-related protein 2					10	3	3.48				
O15143	Actin-related protein 2/3 complex subunit 1B					7	2	0				
P40394	All-trans-retinol dehydrogenase					8	3	1.66				
O43707	Alpha-actinin-4					10	3	16.24				
Q9UBD6	Ammonium transporter Rh type C					7	2	0	M_1-loss			
P07384	Calpain-1 catalytic subunit					3	3	0				
P16152	Carbonyl reductase					7	2	1.67				
P31944	Caspase-14					9	2	3.25				
O00299	Chloride intracellular channel protein 1					17	4	1.86				
Q15517	Corneodesmosin					4	2	4.59				

UniProt-KB Code	Protein name	AD and HC (R)				AD and HC (NR)				NEG (R)		
		Cov. %	Unique Peptides	Score	PTM	Cov. %	Unique Peptides	Score	PTM	Cov. %	Unique Peptides	Score
P01040	Cystatin-A					45	3	2.35				
Q14574	Desmocollin-3					4	3	1.76				
P61803	Dolichyl-diphosphooligosaccharide--protein glycosyltransferase subunit DAD1					19	2	1.98	M_1-loss + NTA			
Q6ZVX7	F-box only protein 50					7	2	0				
P21333	Filamin-A					3	6	0				
P06737	Glycogen phosphorylase, liver form					3	3	0				
P62826	GTP-binding nuclear protein Ran					15	3	1.96	M_1-loss + NTA			
Q6ZN66	Guanylate-binding protein 6					3	2	1.69				
P11142	Heat shock cognate 71 kDa protein					13	4	7.5				
P07900	Heat shock protein HSP 90-alpha					8	2	5.74				
P08238	Heat shock protein HSP 90-beta					8	2	5.61				
P52790	Hexokinase-3					3	3	1.72				
Q9UHA7	Interleukin-36 alpha					14	2	1.73				
O75874	Isocitrate dehydrogenase					6	2	0				
Q9UKR3	Kallikrein-13					12	3	3.37				
P07195	L-lactate dehydrogenase B chain					13	3	26.84				
P09960	Leukotriene A-4 hydrolase					8	4	5.75				
P40926	Malate dehydrogenase, mitochondrial					18	5	7.71				
P60660	Myosin light polypeptide 6					29	3	0				
P43490	Nicotinamide phosphoribosyltransferase					5	3	0				
Q6UX06	Olfactomedin-4					7	3	2.19				

UniProt-KB Code	Protein name	AD and HC (R)				AD and HC (NR)				NEG (R)		
		Cov. %	Unique Peptides	Score	PTM	Cov. %	Unique Peptides	Score	PTM	Cov. %	Unique Peptides	Score
Q96G03	Phosphoglucomutase-2					4	3	3.62				
P18669	Phosphoglycerate mutase 1					13	3	4.18				
P55058	Phospholipid transfer protein					5	2	4.64				
P13797	Plastin-3					10	3	18.45				
Q8WUM4	Programmed cell death 6-interacting protein					4	4	1.76				
Q99623	Prohibitin-2					7	2	0				
Q9UM07	Protein-arginine deiminase type-4					3	2	0				
P61026	Ras-related protein Rab-10					11	2	0				
P62491	Ras-related protein Rab-11A					15	3	0				
P08134	Rho-related GTP-binding protein RhoC					10	2	2.13				
P13489	Ribonuclease inhibitor					5	2	1.69				
P02787	Serotransferrin					4	2	1.72				
Q9UIV8	Serpin B13					20	7	3.89				
P29508	Serpin B3					6	3	6.84				
P22531	Small proline-rich protein 2E					61	3	1.64				
P11169	Solute carrier family 2, facilitated glucose transporter member 3					3	2	0				
O00391	Sulfhydryl oxidase 1					4	3	0				
Q6UWP8	Suprabasin					21	4	6.17				
P10599	Thioredoxin					32	3	7.9				
P07996	Thrombospondin-1					4	4	3.74				
Q86T26	Transmembrane protease serine 11B					4	2	7.8				
P06753	Tropomyosin alpha-3 chain					15	3	2.84				
Q71U36	Tubulin alpha-1A chain					7	3	7.75				
Q13885	Tubulin beta-2A chain					4	2	1.77				

UniProt-KB Code	Protein name	AD and HC (R)				AD and HC (NR)				NEG (R)		
		Cov. %	Unique Peptides	Score	PTM	Cov. %	Unique Peptides	Score	PTM	Cov. %	Unique Peptides	Score
P09769	Tyrosine-protein kinase Fgr					5	2	1.67				
P62987	Ubiquitin-60S ribosomal protein L40					30	3	5.95				
P18206	Vinculin					2	2	0				
O75083	WD repeat-containing protein 1					4	2	0				

*: M_1-loss, Loss of N-terminal methionine; NTA, N-terminal Acetylation

Table S4: Results of the Student t-test for the comparison of the protein LFQ abundancies among AD, HC and NEG groups. Proteins marked in grey were excluded as unspecific interactors according to one or both following conditions: i) higher (↑) or unchanged (ns) LFQ levels in AD and HC groups with respect to NEG (R samples only); ii) a CRAPome score of the found/total ratio over the 25% of total experiments deposited in the database (R and NR samples). For each protein the Uniprot-KB code, the LFQ abundance in in log2 scale expressed as mean of each replicate ± standard deviation (SD), the p value and the fold change calculated by Perseus (in R samples) are reported.

**UniProt-KB Code**	**Protein name**	**Log2 LFQ abundancies (R samples) mean ± SD**			**AD *vs* NEG**		**HC *vs* NEG**		**Enrichment**	**CRAPome Found/Total**
		**AD**	**HC**	**NEG**	**-Log10 p value**	**Fold change**	**-Log10 p value**	**Fold change**		
P52209	6-phosphogluconate dehydrogenase, decarboxylating	24.2 ± 0.2	24.1 ± 0.2	20.4 ± 0.1	3.9	6.1	3.7	3.7	↑AD, ↑HC	122 / 716
O15144	Actin-related protein 2/3 complex subunit 2	18.6 ± 0.4	18.0 ± 1.5	15.5 ± 1.1	3.1	5.8	0.9	2.6	↑AD	71 / 716
P59998	Actin-related protein 2/3 complex subunit 4	19.0 ± 0.3	18.8 ± 1.5	15.3 ± 1.0	3.6	8.0	1.2	3.6	↑AD	111 / 716
P61158	Actin-related protein 3	18.5 ± 0.2	18.3 ± 0.8	15.5 ± 1.2	2.9	2.5	1.3	2.9	↑AD, ↑HC	137 / 716
Q9HDC9	Adipocyte plasma membrane-associated protein	20.2 ± 0.7	20.0 ± 0.9	16.0 ± 1.3	2.9	6.3	1.6	4.0	↑AD, ↑HC	38 / 716
P01009	Alpha-1-antitrypsin	20.0 ± 0.4	19.8 ± 0.3	16.0 ± 1.3	3.2	6.8	1.9	3.8	↑AD, ↑HC	2 / 716
A8K2U0	Alpha-2-macroglobulin-like protein 1	21.0 ± 0.3	21.5 ± 0.3	18.4 ± 0.1	2.3	2.8	3.1	3.0	↑AD, ↑HC	12 / 716
P0DTE7	Alpha-amylase 1B	26.8 ± 0.2	26.1 ± 0.4	23.3 ± 0.1	2.9	5.2	2.4	2.8	↑AD, ↑HC	5 / 716
P04083	Annexin A1	26.5 ± 0.5	26.3 ± 0.7	22.3 ± 0.4	2.3	7.2	2.3	4.1	↑AD, ↑HC	68 / 716
P50995	Annexin A11	19.0 ± 0.4	19.5 ± 0.4	16.1 ± 1.2	2.7	6.2	1.8	3.4	↑AD, ↑HC	55 / 716
P12429	Annexin A3	23.1 ± 0.3	22.6 ± 1.1	19.2 ± 0.5	2.1	4.8	1.5	3.5	↑AD, ↑HC	3 / 716
P08758	Annexin A5	18.9 ± 0.5	18.4 ± 1.1	15.8 ± 1.1	2.8	4.0	1.1	2.6	↑AD	80 / 716
P20292	Arachidonate 5-lipoxygenase-activating protein	21.6 ± 0.3	22.3 ± 0.3	15.9 ± 0.2	3.3	3.5	4.1	6.5	↑AD, ↑HC	0 / 716
P17213	Bactericidal permeability-increasing protein	24.8 ± 0.1	23.6 ± 0.3	21.6 ± 0.0	5.3	5.7	2.5	1.9	↑AD, ↑HC	1 / 716
Q96DR5	BPI fold-containing family A member 2	25.8 ± 0.9	24.1 ± 0.9	21.6 ± 0.3	3.3	2.0	1.5	2.6	↑AD, ↑HC	0 / 716
Q8TDL5	BPI fold-containing family B member 1	23.9 ± 0.1	24.9 ± 0.5	21.9 ± 0.1	3.0	4.2	2.4	3.0	↑AD, ↑HC	6 / 716

UniProt-KB Code	Protein name	Log2 LFQ abundancies (R samples) mean ± SD			AD *vs* NEG		HC *vs* NEG		Enrichment	CRAPome Found/Total
		AD	HC	NEG	-Log10 p value	Fold change	-Log10 p value	Fold change		
Q8N4F0	BPI fold-containing family B member 2	24.3 ± 0.3	23.6 ± 0.3	22.5 ± 0.1	4.2	5.1	1.8	1.2	↑AD	0 / 716
P23280	Carbonic anhydrase 6	24.2 ± 0.2	23.3 ± 0.5	21.7 ± 0.3	2.3	5.3	1.6	1.6	↑AD, ↑HC	1 / 716
P06731	Carcinoembryonic antigen-related cell adhesion molecule 5	19.0 ± 1.3	19.4 ± 0.4	17.3 ± 0.8	0.7	1.7	1.5	2.1	↑HC	0 / 716
P49913	Cathelin-like domain (CLD)	19.4 ± 0.4	18.6 ± 0.6	16.8 ± 0.3	1.4	1.9	1.5	1.8	↑AD, ↑HC	0 / 716
P08311	Cathepsin G	28.1 ± 0.5	27.4 ± 0.4	24.8 ± 0.3	1.3	2.0	2.5	2.6	↑AD, ↑HC	1 / 716
P08962	CD63 antigen	19.5 ± 1.3	19.3 ± 0.7	15.1 ± 0.3	2.8	4.2	2.4	4.2	↑AD, ↑HC	0 / 716
P29373	Cellular retinoic acid-binding protein 2	20.5 ± 0.3	19.7 ± 0.5	14.9 ± 0.8	2.3	3.3	2.4	4.8	↑AD, ↑HC	7 / 716
P0C0L4	Complement C4-A	18.2 ± 1.0	18.8 ± 0.1	15.5 ± 1.0	1.2	2.7	2.1	3.3	↑HC	87 / 716
O75131	Copine-3	17.6 ± 0.2	18.5 ± 0.3	16.2 ± 1.1	1.1	1.4	1.6	2.4	↑HC	91 / 716
Q9UBG3	Cornulin	20.6 ± 2.0	21.0 ± 0.6	14.8 ± 0.7	2.6	5.5	2.7	6.2	↑AD, ↑HC	2 / 716
P31146	Coronin-1A	22.4 ± 0.3	23.1 ± 0.2	15.7 ± 0.8	2.5	3.7	3.4	7.5	↑AD, ↑HC	25 / 716
P04080	Cystatin-B	26.0 ± 0.5	25.1 ± 1.2	19.2 ± 0.2	3.0	9.4	2.2	5.9	↑AD, ↑HC	164 / 716
P01034	Cystatin-C	21.6 ± 0.5	20.8 ± 0.7	19.4 ± 0.1	3.3	3.4	1.2	1.5	↑AD	4 / 716
P28325	Cystatin-D	22.3 ± 0.4	21.1 ± 0.9	15.6 ± 1.0	2.2	3.5	2.2	5.5	↑AD, ↑HC	0 / 716
P09228	Cystatin-SA	18.8 ± 0.8	17.9 ± 0.9	15.2 ± 0.1	3.2	3.4	1.7	2.8	↑AD, ↑HC	1 / 716
P01037	Cystatin-SN	24.5 ± 0.2	23.7 ± 0.7	20.0 ± 0.3	3.1	9.9	2.3	3.7	↑AD, ↑HC	2 / 716
P54108	Cysteine-rich secretory protein 3	21.1 ± 0.5	20.2 ± 0.7	16.0 ± 0.0	2.7	4.7	2.4	4.3	↑AD, ↑HC	0 / 716
P31930	Cytochrome b-c1 complex subunit 1	18.8 ± 0.1	19.2 ± 0.5	15.6 ± 0.6	2.7	3.2	2.3	3.6	↑AD, ↑HC	157 / 716
P32926	Desmoglein-3	20.0 ± 0.2	20.6 ± 0.3	17.6 ± 0.8	3.2	5.1	2.1	3.1	↑AD, ↑HC	0 / 716
Q96HE7	ERO1-like protein alpha	19.2 ± 1.0	19.8 ± 0.3	15.5 ± 2.0	1.2	2.7	1.5	4.3	↑HC	97 / 716
Q16610	Extracellular matrix protein 1	20.3 ± 0.5	20.3 ± 0.4	15.7 ± 0.6	1.8	3.8	2.9	4.6	↑AD, ↑HC	14 / 716
Q01469	Fatty acid-binding protein 5	24.0 ± 0.3	23.8 ± 0.4	16.6 ± 3.5	2.6	2.8	1.5	7.2	↑AD, ↑HC	106 / 716
P02671	Fibrinogen alpha chain	19.7 ± 0.7	19.9 ± 0.4	16.3 ± 1.1	2.8	6.7	1.9	3.6	↑AD, ↑HC	2 / 716
P02675	Fibrinogen beta chain	21.2 ± 0.6	21.3 ± 0.1	15.7 ± 0.6	1.8	3.1	3.2	5.6	↑AD, ↑HC	15 / 716
P02679	Fibrinogen gamma chain	22.1 ± 0.2	22.7 ± 0.3	19.7 ± 0.4	1.7	4.2	2.8	3.1	↑AD, ↑HC	2 / 716
P17931	Galectin-3	19.8 ± 0.7	20.0 ± 0.4	15.5 ± 0.1	1.5	3.0	3.3	4.6	↑AD, ↑HC	20 / 716
P47929	Galectin-7	20.3 ± 0.1	20.9 ± 0.8	15.2 ± 0.3	4.3	7.1	2.6	5.8	↑AD, ↑HC	43 / 716
P06396	Gelsolin	20.7 ± 0.5	20.9 ± 0.5	14.9 ± 0.4	2.5	2.5	3.2	6.0	↑AD, ↑HC	49 / 716

UniProt-KB Code	Protein name	Log2 LFQ abundancies (R samples) mean ± SD			AD *vs* NEG		HC *vs* NEG		Enrichment	CRAPome Found/Total
		AD	HC	NEG	-Log10 p value	Fold change	-Log10 p value	Fold change		
P11413	Glucose-6-phosphate 1-dehydrogenase	20.5 ± 0.1	20.5 ± 0.1	15.8 ± 0.4	3.6	3.1	3.8	4.7	↑AD, ↑HC	89 / 716
P06744	Glucose-6-phosphate isomerase	21.7 ± 0.5	21.7 ± 0.1	16.3 ± 1.0	1.5	3.0	2.7	5.4	↑AD, ↑HC	141 / 716
P15104	Glutamine synthetase	18.1 ± 0.7	17.2 ± 1.7	16.0 ± 0.8	2.5	5.4	0.4	1.2	↑AD	58 / 716
P09211	Glutathione S-transferase P	23.0 ± 0.2	22.7 ± 0.4	20.6 ± 0.1	2.4	3.5	2.3	2.1	↑AD, ↑HC	164 / 716
P28676	Grancalcin	22.6 ± 0.6	23.7 ± 0.3	20.0 ± 0.4	3.3	5.7	3.1	3.8	↑AD, ↑HC	1 / 716
P04899	Guanine nucleotide-binding protein G(i) subunit alpha-2	20.5 ± 0.5	20.7 ± 0.4	16.3 ± 0.3	2.3	6.1	2.9	4.4	↑AD, ↑HC	159 / 716
P04792	Heat shock protein beta-1	22.3 ± 0.9	23.4 ± 0.3	19.0 ± 0.4	2.8	4.2	3.2	4.4	↑AD, ↑HC	128 / 716
P11215	Integrin alpha-M	20.8 ± 1.1	21.5 ± 0.5	17.3 ± 0.8	2.6	4.5	2.3	4.3	↑AD, ↑HC	0 / 716
P05107	Integrin beta-2	21.0 ± 0.8	21.4 ± 0.1	15.8 ± 1.8	3.1	4.1	2.1	5.7	↑AD, ↑HC	0 / 716
P22079	Lactoperoxidase	20.8 ± 0.3	20.4 ± 0.2	16.3 ± 1.8	2.8	5.6	1.6	4.1	↑AD, ↑HC	0 / 716
P30740	Leukocyte elastase inhibitor	23.4 ± 0.1	23.0 ± 0.4	19.2 ± 0.4	3.4	7.5	2.7	3.8	↑AD, ↑HC	17 / 716
P08493	Matrix Gla protein	22.2 ± 0.4	20.6 ± 0.4	16.0 ± 0.8	2.4	3.4	2.5	4.7	↑AD, ↑HC	0 / 716
P14780	Matrix metalloproteinase-9	18.5 ± 0.7	19.2 ± 0.5	15.4 ± 1.2	2.6	2.5	1.8	3.9	↑AD, ↑HC	2 / 716
Q9HC84	Mucin-5B	24.6 ± 0.1	23.8 ± 0.5	20.5 ± 0.6	2.4	3.7	2.2	3.2	↑AD, ↑HC	4 / 716
Q8TAX7	Mucin-7	24.7 ± 0.5	23.6 ± 0.2	16.8 ± 0.4	3.1	1.8	4.2	6.8	↑AD, ↑HC	0 / 716
P24158	Myeloblastin	24.0 ± 0.6	23.4 ± 0.7	21.9 ± 0.1	2.9	2.6	1.2	1.5	↑AD	1 / 716
P41218	Myeloid cell nuclear differentiation antigen	22.5 ± 0.7	22.7 ± 0.7	16.1 ± 1.1	1.5	3.2	2.5	6.6	↑AD, ↑HC	2 / 716
P05164	Myeloperoxidase	26.4 ± 0.2	26.2 ± 0.1	24.6 ± 0.5	3.6	4.5	2.1	1.7	↑AD, ↑HC	3 / 716
P59665	Neutrophil defensin 1	27.7 ± 0.4	28.3 ± 0.6	26.3 ± 0.0	1.8	1.4	1.6	2.0	↑HC	21 / 716
P08246	Neutrophil elastase	28.5 ± 0.6	27.7 ± 0.3	25.6 ± 0.3	3.7	5.8	2.3	2.1	↑AD, ↑HC	2 / 716
O15162	Phospholipid scramblase 1	18.6 ± 0.4	18.6 ± 0.5	15.8 ± 0.0	2.0	4.1	2.2	2.8	↑AD, ↑HC	0 / 716
P13796	Plastin-2	22.6 ± 0.2	22.3 ± 0.1	18.9 ± 0.1	3.5	4.0	4.2	3.5	↑AD, ↑HC	167 / 716
P12273	Prolactin-inducible protein	24.9 ± 0.7	24.5 ± 0.5	22.6 ± 0.4	2.8	6.0	1.8	2.1	↑AD, ↑HC	59 / 716
P06703	Protein S100-A6	20.0 ± 0.8	20.0 ± 0.6	14.9 ± 0.8	3.8	4.8	2.4	5.1	↑AD, ↑HC	72 / 716
P05109	Protein S100-A8	26.7 ± 0.3	26.9 ± 0.5	24.9 ± 0.3	3.5	6.9	1.9	1.9	↑AD, ↑HC	79 / 716
P06702	Protein S100-A9	25.5 ± 0.4	25.0 ± 0.7	22.7 ± 0.3	4.5	3.2	1.6	2.3	↑AD, ↑HC	116 / 716

UniProt-KB Code	Protein name	Log2 LFQ abundancies (R samples) mean ± SD			AD *vs* NEG		HC *vs* NEG		Enrichment	CRAPome Found/Total
		AD	HC	NEG	-Log10 p value	Fold change	-Log10 p value	Fold change		
Q08188	Protein-glutamine gamma-glutamyltransferase E	25.1 ± 0.9	25.1 ± 0.4	22.8 ± 0.4	2.6	3.1	2.2	2.3	↑AD, ↑HC	47 / 716
P50395	Rab GDP dissociation inhibitor beta	19.2 ± 0.5	19.1 ± 0.5	16.2 ± 0.1	1.6	5.4	2.5	2.9	↑AD, ↑HC	166 / 716
P60763	Ras-related C3 botulinum toxin substrate 3	21.8 ± 0.3	21.7 ± 0.4	15.8 ± 1.0	2.0	6.2	2.7	5.9	↑AD, ↑HC	42 / 716
P51159	Ras-related protein Rab-27A	19.7 ± 0.6	20.2 ± 0.7	15.5 ± 0.8	3.7	4.2	2.2	4.7	↑AD, ↑HC	0 / 716
P52566	Rho GDP-dissociation inhibitor 2	20.4 ± 0.7	19.5 ± 0.6	14.9 ± 0.9	1.5	2.3	2.2	4.6	↑AD, ↑HC	4 / 716
P48594	Serpin B4	21.0 ± 0.4	21.0 ± 0.3	14.8 ± 1.0	2.0	2.7	2.8	6.2	↑AD, ↑HC	34 / 716
P36952	Serpin B5	20.0 ± 0.7	20.1 ± 0.5	16.1 ± 1.3	3.0	2.5	1.9	4.0	↑AD, ↑HC	3 / 716
Q9UBC9	Small proline-rich protein 3	22.3 ± 1.2	22.2 ± 0.5	15.8 ± 0.5	3.7	3.7	3.3	6.4	↑AD, ↑HC	4 / 716
P29401	Transketolase	21.0 ± 0.3	21.1 ± 0.4	15.4 ± 0.5	2.8	9.6	3.2	5.7	↑AD, ↑HC	168 / 716
P60174	Triosephosphate isomerase	18.3 ± 0.6	19.7 ± 0.4	17.5 ± 0.1	0.9	0.9	2.4	2.3	↑HC	177 / 716
Q16851	UTP--glucose-1-phosphate uridylyltransferase	19.2 ± 0.3	19.5 ± 0.2	15.9 ± 0.1	2.7	10.2	3.8	3.6	↑AD, ↑HC	14 / 716
Q96DA0	Zymogen granule protein 16 homolog B	26.8 ± 0.3	25.8 ± 0.8	24.4 ± 0.5	3.9	3.8	1.0	1.4	↑AD	11 / 716
P61160	Actin-related protein 2									140 / 716
O15143	Actin-related protein 2/3 complex subunit 1B									44 / 716
P40394	All-trans-retinol dehydrogenase									6 / 716
Q9UBD6	Ammonium transporter Rh type C									0 / 716
P07384	Calpain-1 catalytic subunit									30 / 716
P16152	Carbonyl reductase									143 / 716
P31944	Caspase-14									62 / 716
O00299	Chloride intracellular channel protein 1									147 / 716
Q15517	Corneodesmosin									50 / 716
P01040	Cystatin-A									57 / 716
Q14574	Desmocollin-3									15 / 716

UniProt-KB Code	Protein name	Log2 LFQ abundancies (R samples) mean ± SD			AD *vs* NEG		HC *vs* NEG		Enrichment	CRAPome Found/Total
		AD	HC	NEG	-Log10 p value	Fold change	-Log10 p value	Fold change		
P61803	Dolichyl-diphosphooligosaccharide--protein glycosyltransferase subunit DAD1									27 / 716
Q6ZVX7	F-box only protein 50									21 / 716
P06737	Glycogen phosphorylase, liver form									74 / 716
Q6ZN66	Guanylate-binding protein 6									1 / 716
P52790	Hexokinase-3									23 / 716
Q9UHA7	Interleukin-36 alpha									1 / 716
O75874	Isocitrate dehydrogenase									62 / 716
Q9UKR3	Kallikrein-13									0 / 716
P09960	Leukotriene A-4 hydrolase									66 / 716
P43490	Nicotinamide phosphoribosyltransferase									135 / 716
Q6UX06	Olfactomedin-4									1 / 716
Q96G03	Phosphoglucomutase-2									25 / 716
P55058	Phospholipid transfer protein									0 / 716
Q9UM07	Protein-arginine deiminase type-4									0 / 716
P61026	Ras-related protein Rab-10									176 / 716
P62491	Ras-related protein Rab-11A									125 / 716
P08134	Rho-related GTP-binding protein RhoC									77 / 716
P13489	Ribonuclease inhibitor									70 / 716
P02787	Serotransferrin									11 / 716
Q9UIV8	Serpin B13									1 / 716
P29508	Serpin B3									45 / 716
P22531	Small proline-rich protein 2E									28 / 716
P11169	Solute carrier family 2, facilitated glucose transporter member 3									1 / 716
O00391	Sulfhydryl oxidase 1									1 / 716
Q6UWP8	Suprabasin									68 / 716

UniProt-KB Code	Protein name	Log2 LFQ abundancies (R samples) mean ± SD			AD *vs* NEG		HC *vs* NEG		Enrichment	CRAPome Found/Total
		AD	HC	NEG	-Log10 p value	Fold change	-Log10 p value	Fold change		
P07996	Thrombospondin-1									14 / 716
Q86T26	Transmembrane protease serine 11B									0 / 716
P09769	Tyrosine-protein kinase Fgr									157 / 716
P18206	Vinculin									156 / 716
O75083	WD repeat-containing protein 1									89 / 716
P31946	14-3-3 protein beta/alpha	19.3 ± 0.5	19.4 ± 0.6	15.5 ± 1.3	1.8	3.8	1.8	3.9	↑AD, ↑HC	368 / 716
P61981	14-3-3 protein gamma	18.2 ± 0.6	18.6 ± 0.5	15.3 ± 1.9	1.2	3.0	1.3	3.3	ns	332 / 716
P31947	14-3-3 protein sigma	23.1 ± 0.5	23.0 ± 0.7	15.6 ± 0.4	3.4	7.5	3.1	7.4	↑AD, ↑HC	314 / 716
P63104	14-3-3 protein zeta/delta	23.3 ± 0.3	23.2 ± 0.6	16.2 ± 0.0	4.3	7.1	3.3	7.0	↑AD, ↑HC	410 / 716
P08865	40S ribosomal protein SA	18.9 ± 0.6	19.6 ± 0.4	16.5 ± 1.1	1.3	2.4	1.7	3.1	↑AD, ↑HC	335 / 716
P62906	60S ribosomal protein L10a	17.9 ± 0.8	17.7 ± 0.8	15.9 ± 0.2	1.3	2.0	1.2	1.8	↑AD, ↑HC	280 / 716
P23526	Adenosylhomocysteinase	18.3 ± 0.2	17.2 ± 0.3	15.6 ± 2.9	0.8	2.7	0.4	1.6	ns	232 / 716
Q01518	Adenylyl cyclase-associated protein 1	18.5 ± 1.1	18.8 ± 0.4	15.1 ± 1.9	1.1	3.4	1.4	3.7	↑AD, ↑HC	182 / 716
P61204	ADP-ribosylation factor 3	19.6 ± 0.4	19.2 ± 0.6	16.1 ± 0.7	2.4	3.5	1.9	3.1	↑AD, ↑HC	261 / 716
P05141	ADP/ATP translocase 2	19.5 ± 0.6	19.9 ± 0.7	16.0 ± 0.6	2.2	3.5	2.1	4.0	↑AD, ↑HC	525 / 716
P02768	Albumin	25.7 ± 0.4	25.5 ± 0.2	23.3 ± 0.1	2.5	2.5	3.2	2.3	↑AD, ↑HC	239 / 716
P06733	Alpha-enolase	22.0 ± 0.1	22.8 ± 0.1	20.2 ± 0.1	3.1	1.8	4.2	2.5	↑AD, ↑HC	478 / 716
P07355	Annexin A2	23.7 ± 0.3	23.3 ± 1.0	19.7 ± 0.1	3.5	4.0	1.8	3.6	↑AD, ↑HC	398 / 716
P03973	Antileukoproteinase	26.0 ± 0.7	25.7 ± 0.5	26.0 ± 0.3	0.0	0.0	0.3	-0.3	ns	4 / 716
P02647	Apolipoprotein A-I	17.6 ± 1.6	18.5 ± 0.9	19.7 ± 0.1	0.7	-2.0	0.8	-1.2	ns	17 / 716
P25705	ATP synthase subunit alpha, mitochondrial	21.1 ± 0.2	21.5 ± 0.6	15.6 ± 2.5	1.6	5.4	1.6	5.9	↑AD, ↑HC	496 / 716
P06576	ATP synthase subunit beta, mitochondrial	21.5 ± 0.1	21.3 ± 0.5	15.7 ± 0.1	5.3	5.7	3.3	5.6	↑AD, ↑HC	427 / 716
P20160	Azurocidin	25.5 ± 0.3	24.7 ± 0.6	24.2 ± 0.3	1.8	1.2	0.4	0.5	ns	0 / 716
P12830	Cadherin-1	19.6 ± 0.4	18.4 ± 1.0	19.0 ± 0.2	0.9	0.7	0.3	-0.6	ns	0 / 716
Q14CN2	Calcium-activated chloride channel regulator 4	17.2 ± 0.6	16.7 ± 0.8	16.1 ± 0.5	0.9	1.1	0.4	0.6	ns	0 / 716
P23528	Cofilin-1	21.3 ± 0.2	21.7 ± 0.6	15.2 ± 1.6	2.3	6.1	2.2	6.5	↑AD, ↑HC	496 / 716

UniProt-KB Code	Protein name	Log2 LFQ abundancies (R samples) mean ± SD			AD *vs* NEG		HC *vs* NEG		Enrichment	CRAPome Found/Total
		AD	HC	NEG	-Log10 p value	Fold change	-Log10 p value	Fold change		
P02747	Complement C1q subcomponent subunit C	20.6 ± 0.6	22.1 ± 0.5	21.7 ± 0.4	1.0	-1.1	0.5	0.5	ns	0 / 716
P01024	Complement C3	19.1 ± 0.8	19.2 ± 0.5	18.8 ± 0.8	0.2	0.3	0.3	0.4	ns	45 / 716
Q9UGM3	Deleted in malignant brain tumors 1 protein	26.6 ± 0.4	26.4 ± 0.1	25.1 ± 0.7	1.4	1.5	1.5	1.3	ns	9 / 716
P81605	Dermcidin	21.4 ± 1.5	20.7 ± 0.5	19.9 ± 0.1	0.6	1.5	0.8	0.8	ns	279 / 716
Q02413	Desmoglein-1	17.9 ± 1.9	18.8 ± 0.4	20.8 ± 1.1	0.8	-2.9	1.3	-2.0	↑NEG	116 / 716
P15924	Desmoplakin	21.3 ± 1.4	22.1 ± 0.6	21.7 ± 2.6	0.1	-0.3	0.1	0.4	ns	328 / 716
P68104	Elongation factor 1-alpha 1	22.2 ± 0.5	22.2 ± 0.3	19.1 ± 1.5	1.5	3.2	1.5	3.1	ns	653 / 716
P26641	Elongation factor 1-gamma	16.4 ± 2.1	16.6 ± 0.5	16.7 ± 0.7	0.1	-0.3	0.1	-0.1	ns	461 / 716
P12724	Eosinophil cationic protein	22.1 ± 1.0	22.4 ± 0.8	20.7 ± 1.3	0.6	1.4	0.8	1.7	ns	0 / 716
P15311	Ezrin	21.0 ± 0.7	21.3 ± 0.4	14.8 ± 1.8	2.0	6.2	2.1	6.5	↑HC	264 / 716
P52907	F-actin-capping protein subunit alpha-1	19.0 ± 0.2	18.6 ± 1.3	15.9 ± 1.2	1.8	3.1	1.0	2.6	↑AD	318 / 716
P47756	F-actin-capping protein subunit beta	20.3 ± 0.4	20.4 ± 0.4	15.5 ± 1.3	2.1	4.8	2.1	4.8	↑AD, ↑HC	366 / 716
P04075	Fructose-bisphosphate aldolase A	23.2 ± 0.5	23.0 ± 0.7	18.0 ± 0.4	2.9	5.2	2.6	5.0	↑AD, ↑HC	301 / 716
Q08380	Galectin-3-binding protein	21.6 ± 0.7	21.4 ± 0.4	20.9 ± 0.6	0.5	0.7	0.5	0.5	ns	102 / 716
P04406	Glyceraldehyde-3-phosphate dehydrogenase	23.4 ± 0.2	23.5 ± 0.5	19.9 ± 0.2	3.3	3.5	2.6	3.6	↑AD, ↑HC	458 / 716
P00738	Haptoglobin	20.2 ± 0.4	19.2 ± 1.2	19.8 ± 0.8	0.4	0.5	0.2	-0.5	ns	15 / 716
P0DMV9	Heat shock 70 kDa protein 1B	20.9 ± 0.3	20.9 ± 0.5	19.0 ± 1.7	0.9	1.9	0.9	1.9	ns	698 / 716
P16402	Histone H1.3	21.6 ± 0.6	21.0 ± 0.3	18.5 ± 1.3	1.5	3.0	1.4	2.4	↑AD, ↑HC	600 / 716
P16401	Histone H1.5	21.9 ± 0.0	20.6 ± 0.3	20.0 ± 1.0	1.4	1.9	0.5	0.6	↑AD, ↑HC	243 / 716
Q16778	Histone H2B type 2-E	26.3 ± 0.6	25.2 ± 0.4	16.4 ± 1.1	3.1	9.9	3.1	8.8	↑AD, ↑HC	513 / 716
Q5QNW6	Histone H2B type 2-F	24.8 ± 0.4	23.4 ± 0.6	15.4 ± 1.2	3.0	9.4	2.7	8.0	↑AD, ↑HC	515 / 716
P84243	Histone H3.3	25.5 ± 0.8	24.9 ± 0.3	15.3 ± 1.5	2.7	10.2	2.8	9.6	↑AD, ↑HC	384 / 716
P62805	Histone H4	26.0 ± 0.3	24.9 ± 0.4	21.8 ± 0.4	3.0	4.2	2.5	3.1	↑AD, ↑HC	489 / 716
Q86YZ3	Hornerin	17.6 ± 2.4	18.5 ± 1.9	18.1 ± 1.4	0.1	-0.5	0.1	0.4	ns	223 / 716
P14923	Junction plakoglobin	20.4 ± 1.6	21.2 ± 0.5	21.3 ± 2.1	0.2	-0.9	0.0	0.0	ns	244 / 716
Q9P0G3	Kallikrein-14	19.9 ± 0.2	19.8 ± 0.5	18.8 ± 0.5	1.5	1.2	0.9	1.1	ns	1 / 716

UniProt-KB Code	Protein name	Log2 LFQ abundancies (R samples) mean ± SD			AD *vs* NEG		HC *vs* NEG		Enrichment	CRAPome Found/Total
		AD	HC	NEG	-Log10 p value	Fold change	-Log10 p value	Fold change		
P00338	L-lactate dehydrogenase A chain	24.0 ± 0.2	24.3 ± 0.3	20.8 ± 0.1	3.6	3.1	3.3	3.5	↑AD, ↑HC	304 / 716
P02788	Lactotransferrin	25.2 ± 0.8	26.4 ± 0.2	25.7 ± 0.9	0.2	-0.4	0.7	0.8	ns	61 / 716
P61626	Lysozyme C	24.5 ± 0.4	24.1 ± 1.0	23.9 ± 0.2	1.0	0.7	0.1	0.2	ns	74 / 716
P26038	Moesin	17.9 ± 1.6	18.8 ± 0.5	20.0 ± 0.9	0.7	-2.1	0.8	-1.2	ns	230 / 716
O14950	Myosin regulatory light chain 12B	18.3 ± 1.1	20.1 ± 0.6	15.3 ± 0.2	1.5	3.0	2.8	4.8	↑AD, ↑HC	295 / 716
P35579	Myosin-9	23.1 ± 0.6	24.0 ± 0.4	15.9 ± 1.6	2.3	7.2	2.6	8.1	↑AD, ↑HC	448 / 716
O75594	Peptidoglycan recognition protein 1	18.5 ± 0.2	17.9 ± 0.2	18.8 ± 0.8	0.2	-0.2	0.8	-0.8	ns	0 / 716
Q06830	Peroxiredoxin-1	23.8 ± 0.4	23.3 ± 0.3	14.3 ± 1.5	2.8	9.6	2.8	9.1	↑AD, ↑HC	549 / 716
P32119	Peroxiredoxin-2	19.8 ± 0.8	19.6 ± 0.3	15.6 ± 1.4	1.7	4.2	1.8	4.0	↑AD, ↑HC	482 / 716
Q13162	Peroxiredoxin-4	18.7 ± 0.4	18.3 ± 0.6	15.3 ± 0.5	2.4	3.4	2.0	3.0	↑AD, ↑HC	394 / 716
P30041	Peroxiredoxin-6	20.8 ± 0.6	20.7 ± 0.5	15.5 ± 1.0	2.3	5.2	2.4	5.2	↑AD, ↑HC	388 / 716
P00558	Phosphoglycerate kinase 1	21.0 ± 0.6	21.5 ± 0.3	16.9 ± 1.1	2.0	4.1	2.3	4.7	↑AD, ↑HC	200 / 716
Q13835	Plakophilin-1	18.5 ± 1.3	19.3 ± 0.5	18.1 ± 2.4	0.1	0.4	0.4	1.2	ns	41 / 716
P01833	Polymeric immunoglobulin receptor	26.0 ± 1.1	25.7 ± 0.2	26.1 ± 0.4	0.1	-0.1	0.7	-0.4	ns	12 / 716
P07737	Profilin-1	21.7 ± 0.1	21.3 ± 0.8	15.6 ± 0.4	3.9	6.1	2.5	5.7	↑AD, ↑HC	308 / 716
P35232	Prohibitin	19.6 ± 0.8	19.7 ± 0.4	16.1 ± 2.4	1.1	3.5	1.2	3.6	ns	269 / 716
P60900	Proteasome subunit alpha type-6	18.8 ± 0.3	18.8 ± 0.5	15.9 ± 0.5	2.3	2.8	2.1	2.9	↑AD, ↑HC	193 / 716
P07237	Protein disulfide-isomerase	19.8 ± 0.6	19.9 ± 0.2	16.1 ± 0.2	2.4	3.7	3.6	3.8	↑AD, ↑HC	247 / 716
P14618	Pyruvate kinase PKM	23.9 ± 0.4	24.1 ± 0.2	19.2 ± 0.6	2.7	4.7	3.1	4.9	↑AD, ↑HC	536 / 716
Q96P63	Serpin B12	18.0 ± 1.4	18.9 ± 0.8	18.4 ± 2.1	0.1	-0.4	0.1	0.5	ns	49 / 716
P0DPH8	Tubulin alpha-3D chain	19.8 ± 0.6	20.3 ± 0.1	16.5 ± 0.0	2.3	3.3	5.0	3.8	↑AD, ↑HC	690 / 716
P08670	Vimentin	17.9 ± 0.6	19.3 ± 0.3	15.6 ± 0.8	1.5	2.3	2.4	3.7	↑AD, ↑HC	543 / 716
P21796	Voltage-dependent anion-selective channel protein 1	18.4 ± 0.3	18.2 ± 1.3	15.7 ± 0.8	2.0	2.7	1.0	2.5	↑AD, ↑HC	213 / 716
P45880	Voltage-dependent anion-selective channel protein 2	19.3 ± 0.2	19.5 ± 1.1	15.7 ± 0.7	2.5	3.7	1.6	3.8	↑AD, ↑HC	256 / 716
P30050	60S ribosomal protein L12	16.3 ± 0.3	16.6 ± 0.6	18.3 ± 0.6	1.8	-2.0	1.3	-1.7	↑NEG	397 / 716
P60709	Actin, cytoplasmic 1	16.5 ± 0.9	16.8 ± 1.1	24.0 ± 0.1	2.8	-7.5	2.5	-7.2	↑NEG	667 / 716
P12814	Alpha-actinin-1	16.4 ± 0.9	16.7 ± 0.6	17.8 ± 0.2	0.9	-1.4	1.1	-1.1	ns	314 / 716

UniProt-KB Code	Protein name	Log2 LFQ abundancies (R samples) mean ± SD			AD *vs* NEG		HC *vs* NEG		Enrichment	CRAPome Found/Total
		AD	HC	NEG	-Log10 p value	Fold change	-Log10 p value	Fold change		
P05089	Arginase-1	16.7 ± 0.9	17.1 ± 0.3	19.8 ± 1.2	1.3	-3.0	1.6	-2.7	↑NEG	57 / 716
Q9NP55	BPI fold-containing family A member 1	16.0 ± 0.5	16.3 ± 0.4	17.3 ± 0.8	1.0	-1.3	0.8	-1.0	ns	3 / 716
Q9NZT1	Calmodulin-like protein 5	17.2 ± 0.6	17.5 ± 0.6	17.8 ± 1.1	0.3	-0.5	0.1	-0.2	ns	150 / 716
P04040	Catalase	16.9 ± 0.4	17.2 ± 0.7	18.9 ± 1.3	1.1	-2.0	0.9	-1.7	ns	57 / 716
Q08554	Desmocollin-1	16.7 ± 0.7	17.0 ± 0.0	20.1 ± 1.8	1.3	-3.4	1.3	-3.1	↑NEG	71 / 716
P13639	Elongation factor 2	16.5 ± 1.6	16.8 ± 1.6	15.8 ± 3.0	0.1	0.7	0.2	1.0	ns	488 / 716
Q5D862	Filaggrin-2	16.5 ± 0.7	16.8 ± 0.4	17.1 ± 1.2	0.2	-0.5	0.1	-0.2	ns	153 / 716
Q9H4G4	Golgi-associated plant pathogenesis-related protein 1	16.8 ± 0.6	17.1 ± 0.8	17.6 ± 0.8	0.5	-0.7	0.3	-0.5	ns	7 / 716
Q16777	Histone H2A type 2-C	16.3 ± 1.4	16.7 ± 1.0	24.9 ± 0.4	2.4	-8.5	2.8	-8.2	↑NEG	552 / 716
P23284	Peptidyl-prolyl cis-trans isomerase B	16.1 ± 1.0	16.6 ± 0.4	16.8 ± 0.7	0.3	-0.7	1.3	-1.2	ns	228 / 716
Q6P4A8	Phospholipase B-like 1	16.3 ± 0.5	16.6 ± 0.4	17.8 ± 0.4	1.4	-1.5	1.3	-1.2	↑NEG	4 / 716
P28799	Progranulin	17.0 ± 0.8	17.3 ± 0.3	20.0 ± 0.1	1.8	-3.0	3.0	-2.7	↑NEG	16 / 716
P22735	Protein-glutamine gamma-glutamyltransferase K	16.6 ± 0.7	16.9 ± 1.2	18.1 ± 1.1	0.8	-1.5	0.5	-1.2	ns	35 / 716
O43707	Alpha-actinin-4									335 / 716
P21333	Filamin-A									508 / 716
P62826	GTP-binding nuclear protein Ran									440 / 716
P11142	Heat shock cognate 71 kDa protein									703 / 716
P07900	Heat shock protein HSP 90-alpha									565 / 716
P08238	Heat shock protein HSP 90-beta									573 / 716
P07195	L-lactate dehydrogenase B chain									340 / 716
P40926	Malate dehydrogenase, mitochondrial									198 / 716
P60660	Myosin light polypeptide 6									411 / 716
P18669	Phosphoglycerate mutase 1									239 / 716
P13797	Plastin-3									208 / 716
Q8WUM4	Programmed cell death 6-interacting protein									227 / 716
Q99623	Prohibitin-2									243 / 716
P10599	Thioredoxin									432 / 716

UniProt-KB Code	Protein name	Log2 LFQ abundancies (R samples) mean ± SD			AD *vs* NEG		HC *vs* NEG		Enrichment	CRAPome Found/Total
		AD	HC	NEG	-Log10 p value	Fold change	-Log10 p value	Fold change		
P06753	Tropomyosin alpha-3 chain									354 / 716
Q71U36	Tubulin alpha-1A chain									694 / 716
Q13885	Tubulin beta-2A chain									675 / 716
P62987	Ubiquitin-60S ribosomal protein L40									462 / 716

Table S5: Qualitative comparison of the proteins identified in CoIPs from AD and HC analyzed under R and NR conditions, after the application of the exclusion criteria. For each protein the Uniprot-KB code is reported, the symbol ● indicates whether the protein was identified under R, NR or both conditions.

**UniProt-KB Code**	**Protein name**	**AD and HC (R)**	**AD and HC (NR)**
P52209	6-phosphogluconate dehydrogenase, decarboxylating	●	●
O15144	Actin-related protein 2/3 complex subunit 2	●	●
P59998	Actin-related protein 2/3 complex subunit 4	●	●
P61158	Actin-related protein 3	●	●
Q9HDC9	Adipocyte plasma membrane-associated protein	●	●
P01009	Alpha-1-antitrypsin	●	●
A8K2U0	Alpha-2-macroglobulin-like protein 1	●	●
P0DTE7	Alpha-amylase 1B	●	●
P04083	Annexin A1	●	●
P50995	Annexin A11	●	●
P12429	Annexin A3	●	●
P08758	Annexin A5	●	
P20292	Arachidonate 5-lipoxygenase-activating protein	●	●
P17213	Bactericidal permeability-increasing protein	●	●
Q96DR5	BPI fold-containing family A member 2	●	●
Q8TDL5	BPI fold-containing family B member 1	●	●
Q8N4F0	BPI fold-containing family B member 2	●	●
P23280	Carbonic anhydrase 6	●	●
P06731	Carcinoembryonic antigen-related cell adhesion molecule 5	●	
P49913	Cathelin-like domain	●	●
P08311	Cathepsin G	●	●
P08962	CD63 antigen	●	●
P29373	Cellular retinoic acid-binding protein 2	●	●
P0C0L4	Complement C4-A	●	●
O75131	Copine-3	●	
Q9UBG3	Cornulin	●	●
P31146	Coronin-1A	●	●
P04080	Cystatin-B	●	●
P01034	Cystatin-C	●	●
P28325	Cystatin-D	●	●
P09228	Cystatin-SA	●	●
P01037	Cystatin-SN	●	●
P54108	Cysteine-rich secretory protein 3	●	●
P31930	Cytochrome b-c1 complex subunit 1	●	
P32926	Desmoglein-3	●	●
Q96HE7	ERO1-like protein alpha	●	●
Q16610	Extracellular matrix protein 1	●	●
Q01469	Fatty acid-binding protein 5	●	●
P02671	Fibrinogen alpha chain	●	●
P02675	Fibrinogen beta chain	●	●
P02679	Fibrinogen gamma chain	●	●
P17931	Galectin-3	●	●

UniProt-KB Code	Protein name	AD and HC (R)	AD and HC (NR)
P47929	Galectin-7	●	●
P06396	Gelsolin	●	●
P11413	Glucose-6-phosphate 1-dehydrogenase	●	●
P06744	Glucose-6-phosphate isomerase	●	●
P15104	Glutamine synthetase	●	
P09211	Glutathione S-transferase P	●	●
P28676	Grancalcin	●	●
P04899	Guanine nucleotide-binding protein G(i) subunit alpha-2	●	●
P04792	Heat shock protein beta-1	●	●
P11215	Integrin alpha-M	●	●
P05107	Integrin beta-2	●	●
P22079	Lactoperoxidase	●	●
P30740	Leukocyte elastase inhibitor	●	●
P08493	Matrix Gla protein	●	●
P14780	Matrix metalloproteinase-9	●	
Q9HC84	Mucin-5B	●	●
Q8TAX7	Mucin-7	●	●
P24158	Myeloblastin	●	●
P41218	Myeloid cell nuclear differentiation antigen	●	●
P05164	Myeloperoxidase	●	●
P59665	Neutrophil defensin 1	●	●
P08246	Neutrophil elastase	●	●
O15162	Phospholipid scramblase 1	●	
P13796	Plastin-2	●	●
P12273	Prolactin-inducible protein	●	●
P06703	Protein S100-A6	●	●
P05109	Protein S100-A8	●	●
P06702	Protein S100-A9	●	●
Q08188	Protein-glutamine gamma-glutamyltransferase E	●	●
P50395	Rab GDP dissociation inhibitor beta	●	●
P60763	Ras-related C3 botulinum toxin substrate 3	●	
P51159	Ras-related protein Rab-27A	●	
P52566	Rho GDP-dissociation inhibitor 2	●	●
P48594	Serpin B4	●	
P36952	Serpin B5	●	●
Q9UBC9	Small proline-rich protein 3	●	●
P29401	Transketolase	●	●
P60174	Triosephosphate isomerase	●	●
Q16851	UTP--glucose-1-phosphate uridylyltransferase	●	●
Q96DA0	Zymogen granule protein 16 homolog B	●	●
P61160	Actin-related protein 2		●
O15143	Actin-related protein 2/3 complex subunit 1B		●
P40394	All-trans-retinol dehydrogenase		●
Q9UBD6	Ammonium transporter Rh type C		●
P07384	Calpain-1 catalytic subunit		●
P16152	Carbonyl reductase		●

UniProt-KB Code	Protein name	AD and HC (R)	AD and HC (NR)
P31944	Caspase-14		•
O00299	Chloride intracellular channel protein 1		•
Q15517	Corneodesmosin		•
P01040	Cystatin-A		•
P61803	Dolichyl-diphosphooligosaccharide--protein glycosyltransferase subunit DAD1		•
Q14574	Desmocollin-3		•
Q6ZVX7	F-box only protein 50		•
P06737	Glycogen phosphorylase, liver form		•
Q6ZN66	Guanylate-binding protein 6		•
P52790	Hexokinase-3		•
Q9UHA7	Interleukin-36 alpha		•
O75874	Isocitrate dehydrogenase		•
Q9UKR3	Kallikrein-13		•
P09960	Leukotriene A-4 hydrolase		•
P43490	Nicotinamide phosphoribosyltransferase		•
Q6UX06	Olfactomedin-4		•
Q96G03	Phosphoglucomutase-2		•
P55058	Phospholipid transfer protein		•
Q9UM07	Protein-arginine deiminase type-4		•
P61026	Ras-related protein Rab-10		•
P62491	Ras-related protein Rab-11A		•
P08134	Rho-related GTP-binding protein RhoC		•
P13489	Ribonuclease inhibitor		•
P02787	Serotransferrin		•
Q9UIV8	Serpin B13		•
P29508	Serpin B3		•
P22531	Small proline-rich protein 2E		•
P11169	Solute carrier family 2, facilitated glucose transporter member 3		•
O00391	Sulfhydryl oxidase 1		•
Q6UWP8	Suprabasin		•
P07996	Thrombospondin-1		•
Q86T26	Transmembrane protease serine 11B		•
P09769	Tyrosine-protein kinase Fgr		•
P18206	Vinculin		•
O75083	WD repeat-containing protein 1		•

Figure S1: SDS-PAGE under NR and R conditions of IP proteins after cystatin B Co-IP assay from AD and HC pools in triplicate (A) and SDS-PAGE under R condition of IP proteins after normal mouse Co-IP assay from NEG pool in duplicate (B). Black lines indicate how each lane has been cut prior to tryptic digestion and nano-HPLC-HR-ESI-MS/MS analysis.

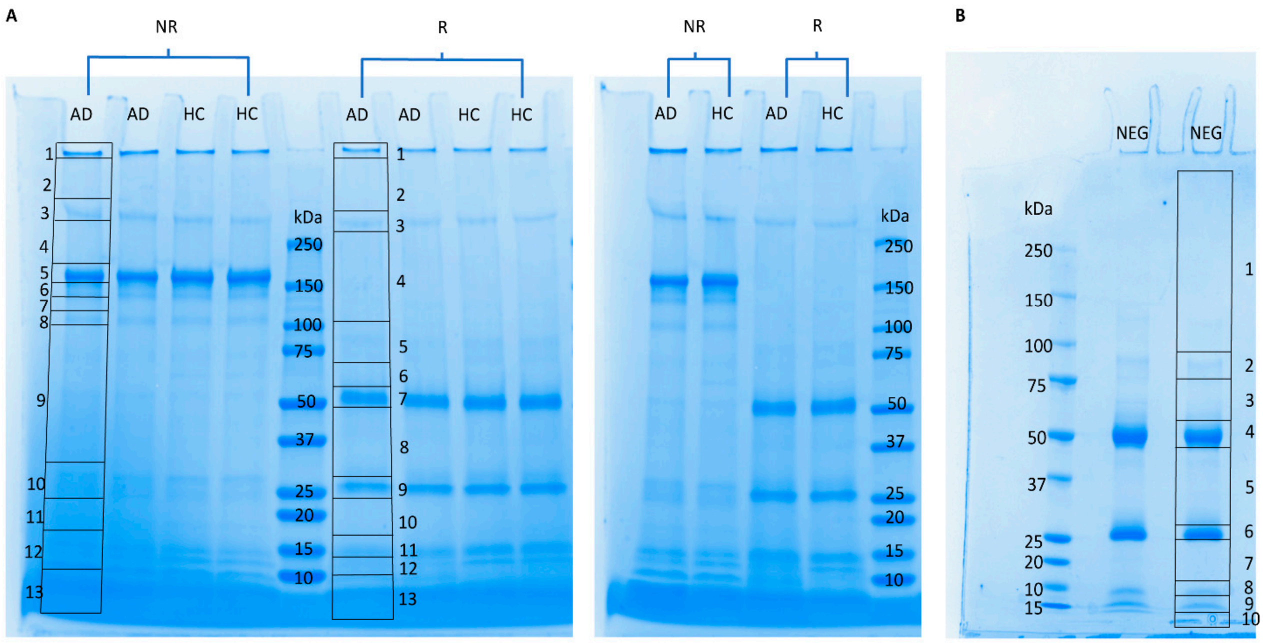

Figure S2: Volcano Plots of significant enriched proteins in AD (blue dots) with respect to NEG (A) and significant enriched proteins in HC (green dots) with respect to NEG (B). In both panels red dots represent enriched proteins in NEG, while grey dots represent unchanged proteins.

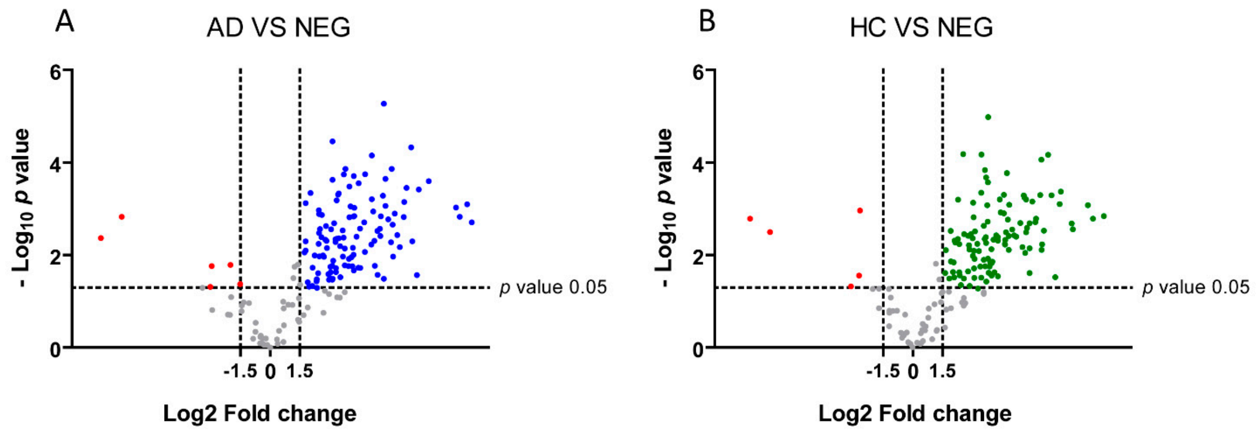

Figure S3: Chart of comparison of the total protein LFQ abundances (Log10) determined by PD software in AD and HC R sample (triplicates) without (panel A), and with (panel B) normalization with respect to the total peptide amount measured in the same samples. The central line represents the variance related to each replicate. SD deviation is indicated. The protein outliers are indicated with black squares.

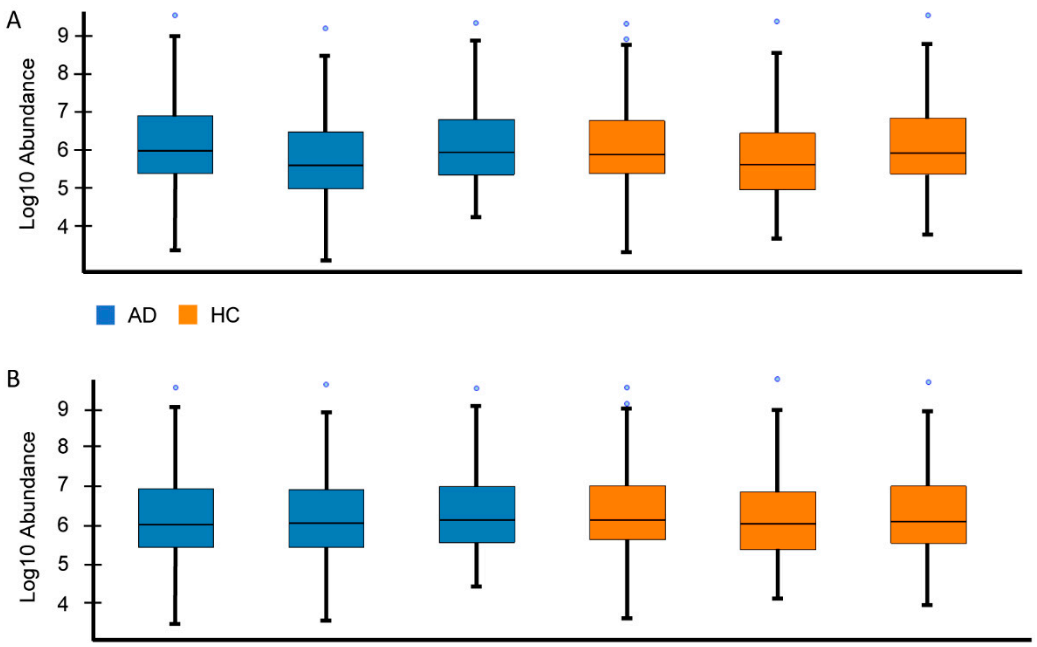

Supplement: Supplementary file 1 [file life-13-00748-s001.zip › Supplementary_file S1_Revised_cleaned.pdf]
